# A Gammaherpesvirus Cooperates with Interferon-alpha/beta-Induced IRF2 to Halt Viral Replication, Control Reactivation, and Minimize Host Lethality

**DOI:** 10.1371/journal.ppat.1002371

**Published:** 2011-11-17

**Authors:** Pratyusha Mandal, Bridgette E. Krueger, Darby Oldenburg, Katherine A. Andry, R. Suzanne Beard, Douglas W. White, Erik S. Barton

**Affiliations:** 1 Department of Biological Sciences, Purdue University, West Lafayette, Indiana, United States of America; 2 Department of Microbiology and Immunology, Wake Forest University School of Medicine, Winston-Salem, North Carolina, United States of America; 3 Department of Health Professions, University of Wisconsin La Crosse, La Crosse, Wisconsin, United States of America; 4 Rheumatology Research Laboratory, Gundersen Lutheran Medical Center, La Crosse, Wisconsin, United States of America; 5 Department of Microbiology, University of Wisconsin La Crosse, La Crosse, Wisconsin, United States of America; University of California at Los Angeles, United States of America

## Abstract

The gammaherpesviruses, including Epstein-Barr virus (EBV) and Kaposi's sarcoma-associated herpesvirus (KSHV), establish latency in memory B lymphocytes and promote lymphoproliferative disease in immunocompromised individuals. The precise immune mechanisms that prevent gammaherpesvirus reactivation and tumorigenesis are poorly defined. Murine gammaherpesvirus 68 (MHV68) is closely related to EBV and KSHV, and type I (alpha/beta) interferons (IFNαβ) regulate MHV68 reactivation from both B cells and macrophages by unknown mechanisms. Here we demonstrate that IFNβ is highly upregulated during latent infection, in the absence of detectable MHV68 replication. We identify an interferon-stimulated response element (ISRE) in the MHV68 M2 gene promoter that is bound by the IFNαβ-induced transcriptional repressor IRF2 during latency *in vivo*. The M2 protein regulates B cell signaling to promote establishment of latency and reactivation. Virus lacking the M2 ISRE (ISREΔ) overexpresses M2 mRNA and displays uncontrolled acute replication *in vivo*, higher latent viral load, and aberrantly high reactivation from latency. These phenotypes of the ISREΔ mutant are B-cell-specific, require IRF2, and correlate with a significant increase in virulence in a model of acute viral pneumonia. We therefore identify a mechanism by which a gammaherpesvirus subverts host IFNαβ signaling in a surprisingly cooperative manner, to directly repress viral replication and reactivation and enforce latency, thereby minimizing acute host disease. Since we find ISREs 5′ to the major lymphocyte latency genes of multiple rodent, primate, and human gammaherpesviruses, we propose that cooperative subversion of IFNαβ-induced IRFs to promote latent infection is an ancient strategy that ensures a stable, minimally-pathogenic virus-host relationship.

## Author Summary

Herpesviruses establish life-long infection in a non-replicating state termed latency. During immune compromise, herpesviruses can reactivate and cause severe disease, including cancer. We investigated mechanisms by which interferons alpha/beta (IFNαβ), a family of antiviral immune genes, inhibit reactivation of murine gammaherpesvirus 68 (MHV68). MHV68 is related to Epstein-Barr virus and Kaposi's sarcoma-associated herpesvirus, human gammaherpesviruses associated with multiple cancers. We made the surprising discovery that during latency, MHV68 cooperates with IFNαβ to inhibit its own replication. Specifically, a viral gene required for reactivation has evolved to be directly repressed by an IFNαβ-induced transcription factor, IRF2. Once virus replication has triggered sufficient IFNαβ production, expression of this viral gene is reduced and reactivation efficiency decreases. This strategy safeguards the health of the host, since a mutant virus that cannot respond to IRF2 replicates uncontrollably and is more virulent. Viral sensing of IFNαβ is also potentially subversive, since it allows MHV68 to detect periods of localized immune quiescence during which it can reactivate and spread to a new host. Thus, we highlight a novel path of virus-host coevolution, toward cooperative subversion of the antiviral immune response. These observations may illuminate new targets for drugs to inhibit herpesvirus reactivation or eliminate herpesvirus-associated tumors.

## Introduction

The gammaherpesviruses (γHVs) establish life-long latent infection in memory B lymphocytes. The human γHVs Epstein-Barr virus (EBV) and Kaposi's sarcoma-associated herpesvirus (KSHV) are the causes of infectious mononucleosis and Kaposi's sarcoma (KS), respectively [1], [2]. γHV latency is a cofactor in the development of lymphomas, sarcomas, and carcinomas. Viral reactivation and neoplasms increase in immune compromised individuals, highlighting the need for immune surveillance to prevent severe disease [2]. Mechanisms of immune control of latent EBV and KSHV are not completely understood due to their human-specific host range. Murine gammaherpesvirus 68 (MHV68) is closely related to the human γHVs and provides a genetic model to study γHV-immune interactions that regulate pathogenesis [3], [4].

We previously uncovered an unexpected role for type I (alpha/beta) interferons (IFNαβ) during MHV68 latency [5]. IFNαβ are a family of antiviral cytokines whose expression is triggered by cellular sensors of viral nucleic acid that activate interferon regulatory factor (IRF) family transcription factors [6]. IRFs bind to interferon stimulated response elements (ISREs) in IFN gene promoters to trigger expression of IFNαβ. IFNαβ signaling via its heterodimeric receptor (IFNAR1/2) induces a large family of interferon-stimulated genes (ISGs) that inhibit viral replication by multiple mechanisms. Once virus infection has been cleared, the IFNαβ-induced transcriptional repressor IRF2 exerts a negative feedback role to terminate IFNαβ expression and prevent inflammatory pathology [7]. Many viruses antagonize IFNαβ expression or ISG function to maximize replication [8]. However, the interactions between latent viruses and IFNαβ are largely unexplored.

We found that mice lacking the IFNαβ receptor (IFNAR1-/-) exhibit increased MHV68 reactivation from latency in both splenic B cells and peritoneal macrophages [5]. This was unexpected since viral molecules that trigger IFNαβ production should be largely absent during latency, when infectious virus is undetectable using classical virologic assays. In addition, known antiviral functions of IFNαβ are critical during acute viral infection, but are thought to be dispensable once replication is controlled [6]. One clue to the mechanism of IFNαβ function during MHV68 latency came from our observation that the MHV68 latent gene M2 is specifically upregulated in splenocytes from IFNAR1-/- mice [5]. M2 is required for establishment of latency in splenic B cells following mucosal infection and is essential for reactivation from B cells [9]. While the precise function of M2 is not known, it interacts with B cell signaling molecules including fyn and vav1, resulting in efficient entry of infected B cells into a germinal center (GC) reaction [10]–[13]. This suggests that M2 is a functional analog of the human γHV B cell signaling mimics LMP2A and K1 of EBV and KSHV, respectively [14]. M2 also promotes differentiation into plasma B cells, the main cell type that supports reactivation of MHV68, EBV, and KSHV [15]. Thus, M2 plays important roles in both establishment of latency and reactivation. Upregulation of M2 in IFNAR1-/- mice suggested that latency and reactivation are directly regulated by IFNαβ-dependent modulation of M2 expression.

Here we show that latent MHV68 infection triggers sustained, IFNαβ-driven expression of IRF2, which binds an ISRE present in the M2 promoter. A mutant virus lacking the M2 ISRE (ISREΔ) exhibits uncontrolled replication and increased host lethality late in acute infection. During latency, ISREΔ overexpresses M2 mRNA, and displays increased viral load and aberrantly high reactivation. These phenotypes were absent in mice lacking B cells, IRF2, or IFNAR1. Thus, we demonstrate that MHV68 subverts IFNαβ-dependent IRF2 signaling to silence expression of a viral B cell signaling mimic, thereby preventing viral replication and reactivation. This demonstrates that viral promoters can cooperate with IFNαβ-induced host transcription factors to directly mediate the antiviral effects of IFNαβ. To our knowledge, this is the first example of viral cooperation with the IFNαβ system. We hypothesize that evolution of IFNαβ-responsive viral promoters provides a selective advantage, by curtailing replication and expansion of the latently-infected reservoir prior to severe host pathology, and by ensuring that reactivation occurs only when the microenvironment of the latent cell favors productive replication. Given the conservation of ISREs in latent promoters of EBV and KSHV [16]–[18], we propose that this cooperative approach is a general regulatory strategy that arose during γHV-host coevolution.

## Results

### IRF2 binds the M2 ISRE during latency *in vivo*

**Figure 1:**
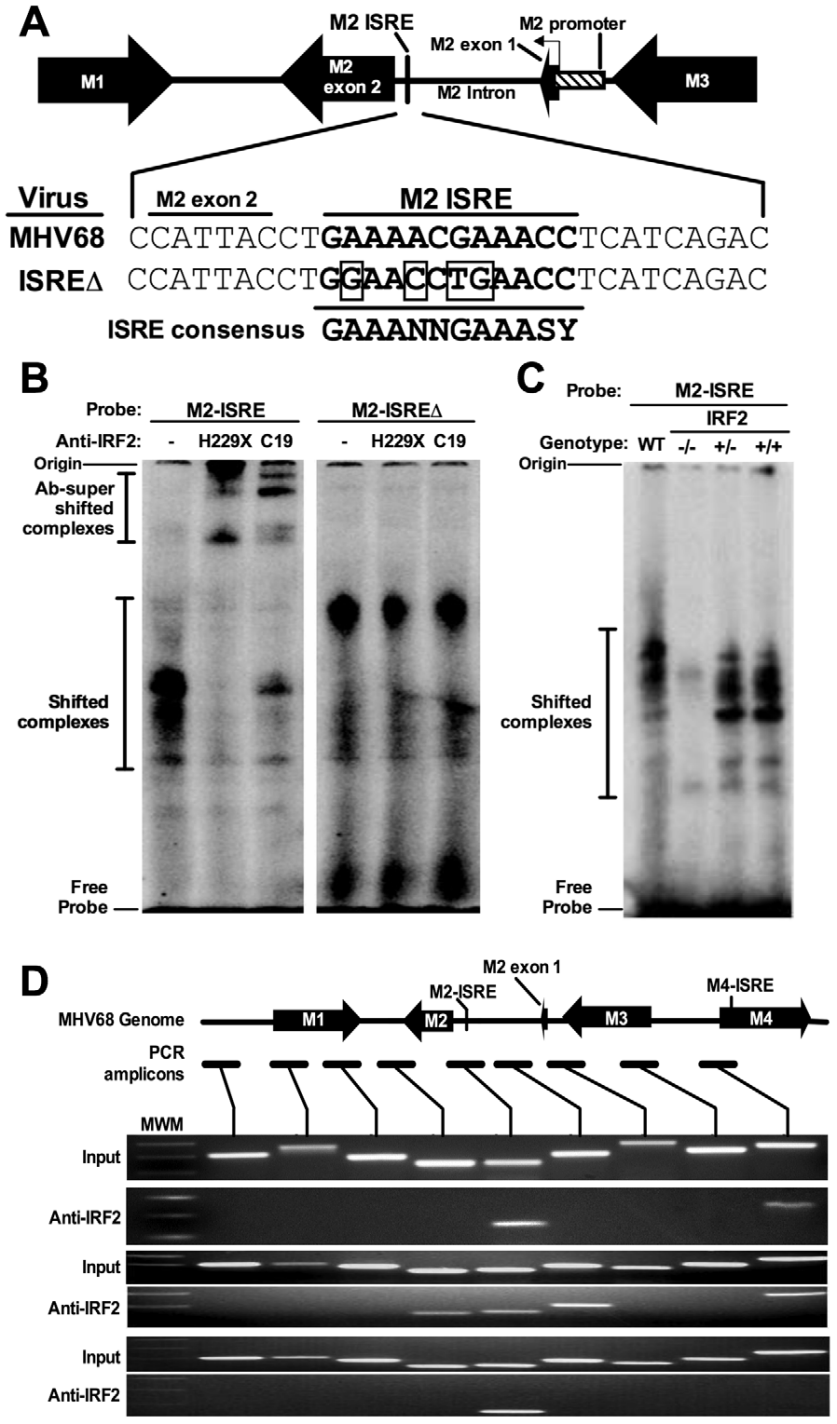
The M2 ISRE binds IRF2 *in vivo* during latency. **(A)** The M2 locus indicating the location of the consensus ISRE. M2 ISRE sequence (nt. 4603-4632 of GenBank Accession U97553.2) and ISREΔ mutations (boxed bases) are shown. Consensus ISRE sequence is based on reference [21]. The location of the basal M2 promoter is based on reference [20]. **(B)** Nuclear extracts from splenocytes of latently-infected C57BL6/J mice were incubated with radiolabeled M2 ISRE or M2 ISREΔ probes to detect M2 ISRE binding proteins via EMSA (shifted complexes). Two different antisera against IRF2 (H229X, C19) were used to detect complexes containing IRF2 (Ab-supershifted complexes). See Figure S1, which demonstrates specificity of complexes bound to M2 ISRE probe. **(C)** Nuclear extracts from splenocytes of uninfected IRF2-/-, IRF2+/- or IRF2+/+ littermates were used in EMSA. WT, nuclear extract from latently-infected C57BL6/J splenocytes. **(D)** Splenocytes harvested from latently-infected 129S2 mice were used to detect IRF2 binding to the M2 ISRE via ChIP. Antiserum against IRF2 was used to precipitate crosslinked, sheared chromatin, which was subjected to PCR amplification. Relative locations of the M2 ISRE PCR amplicon and control amplicons are shown. Input control PCR reactions were performed with 10% total chromatin removed prior to immunoprecipitation. Control precipitations performed without anti-IRF2 antibody yielded no amplicons for any primer set (not shown). Shown are results from three independent experiments using pooled splenocytes from three to five mice per experiment. MWM, molecular weight marker.

We found a consensus ISRE in the M2 intron (Figure 1A). Functional intronic ISREs have been reported, suggesting that this ISRE regulates the M2 promoter [19], [20]. To determine whether the M2 ISRE binds host IRFs, we incubated M2 ISRE probes with nuclear proteins from splenocytes of latently-infected mice in electromobility shift assays (EMSA). As a control, we mutated four residues essential for IRF binding (ISREΔ, Figure 1A) [21]. M2 ISRE and M2 ISREΔ probes formed distinct complexes with nuclear proteins (Figure 1B). Only complexes formed with M2 ISRE were specific, since formation was inhibited with excess unlabeled M2 ISRE but not M2 ISREΔ probe (Figure S1). Two different antisera specific for IRF2 super-shifted M2 ISRE-bound complexes but not those bound to M2 ISREΔ (Figure 1B). IRF2 is an essential component of these complexes, since they are not formed using nuclear extracts from IRF2-/- mice (Figure 1C).

To determine whether IRF2 binds to M2 ISRE *in vivo* we used chromatin immunoprecipitation (ChIP) from splenocytes of latently-infected mice (Figure 1D). Anti-IRF2 antisera enriched DNA within one kilobase of the M2 ISRE, but not adjacent control regions. Interestingly, in two of three experiments, we also detected IRF2 binding to a region in the nearby M4 gene. Analysis of this region revealed a second consensus ISRE (M4-ISRE, Figure 1D) supporting the specificity of the assay. Thus, IRF2 binds the M2 ISRE during latent infection in the spleen.

### IFNβ and IRF2 are induced during MHV68 lytic and latent infection

**Figure 2:**
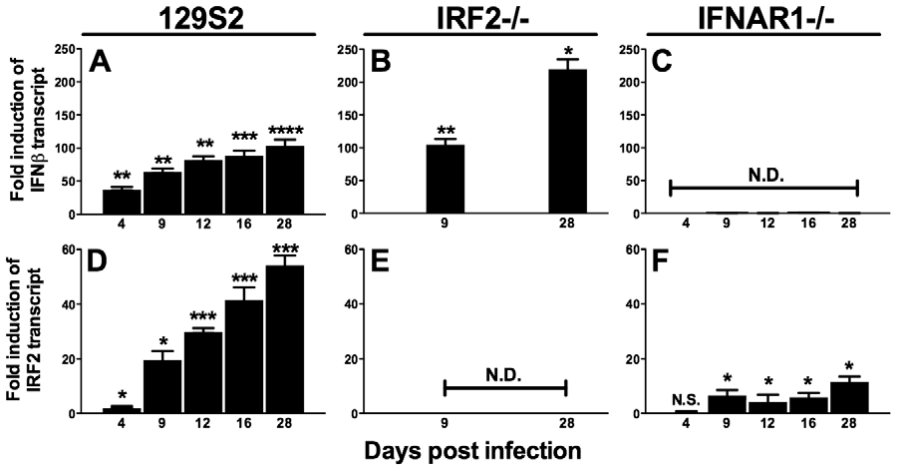
IFNβ and IRF2 are upregulated during acute infection and latency *in vivo*. Total RNA was harvested from splenocytes of mice infected with MHV68 at the indicated times post infection. Quantitative RT-PCR was used to detect spliced transcripts of IFNβ **(A–C)** or IRF2 **(D–F)**. Indicated are the mouse genotypes from which RNA was harvested: 129S2, IRF2-/- (C57BL6/J background), IFNAR1-/- (129S2 background). Expression of IFNβ or IRF2 transcripts is shown as fold induction relative to background-matched uninfected wildtype mice at the same time post infection. Expression in 129S2 and IFNAR1-/- mice is normalized to uninfected 129S2 mice and expression in IRF2-/- mice is normalized to uninfected IRF2+/+ littermates. Shown are mean (+/- SEM) from three pooled independent experiments with two to three mice per group. *p≤ 0.05, **p≤ 0.01, ***p≤ 0.001, ****p≤ 0.0001 by paired, two-tailed t-test. For **A** and **D**, p values represent comparison of transcript levels to uninfected 129S2 mice. For **B** and **F**, p values represent comparison of transcript levels to infected IRF2+/+ littermates or infected 129S2 mice, respectively, at the same time points. N.S., nonspecific (p>0.05). N.D., not detected.

IRF2 is generally a transcriptional repressor, is constitutively expressed at low levels in many cell types including lymphocytes, and is upregulated by IFNαβ [21], [22]. IFNAR1-/- mice display increased reactivation and upregulation of M2 [5], suggesting that IFNαβinduces IRF2-dependent repression of M2 during latency. However, others have reported that IFNαβproteins are not detectable during acute MHV68 infection in the lung [23]. Therefore, we determined kinetics of IFNβand IRF2 expression during MHV68 infection in the spleen of wildtype, IFNAR1-/-, and IRF2-/- mice. Under these conditions, IRF2-/- mice experience no lethality, clear acute infection, and establish latency with no evidence of persistent lytic replication (not shown and Table 1). IFNβ and IRF2 transcripts were strongly induced in a time-dependent fashion during acute infection (Figure 2A,D). Both transcripts were more highly induced during latent infection (16–28 days post infection (dpi)) than at the peak of acute infection (4–9 dpi). Full induction of both transcripts required IFNAR1 (Figure 2C,F), confirming that extracellular IFNαβ proteins are produced and functional. Consistent with the repressive role of IRF2, IFNβ was significantly elevated during latent infection in IRF2-/- mice (Figure 2B). These data demonstrate sustained expression of IFNβ and IRF2 at the major site of MHV68 latency.

**Table 1:** Frequency of latently-infected cells and efficiency of reactivation.

Days post infection	Mouse genotype^1^	Virus	Frequency of latent viral genome^2^		Frequency of latent virus reactivation^2^		Fold increase in reactivation^3^		P^4^		Efficiency of reactivation^5^	
			Spleen	PEC	Spleen	PEC	Spleen	PEC	Spleen	PEC	Spleen	PEC
16	129S2	MHV68	1∶1405	1∶715	1∶233,169	1∶44,052	1	1			.006	.02
	129S2	ISREΔ	1∶1360	1∶623	1∶72,159	1∶39,100	3.2	1.1	.009	N.S.	.018	.015
	IFNAR1-/-	MHV68	1∶501	1∶290	1∶8319	1∶950	28	46			.06	.3
	IFNAR1-/-	ISREΔ	1∶333	1∶378	1∶4100	1∶853	57	52	N.S.	N.S.	.09	.41
*Key conclusions: M2 ISRE regulates reactivation from splenocytes, but not peritoneal cells, during early latency. M2 ISRE does not regulate reactivation in the absence of IFNAR1.*												
28	129S2	MHV68	1∶658	1∶611	1∶265,580	1∶25,262	1	1			.002	.02
	129S2	ISREΔ	1∶327	1∶632	1∶72,185	1∶24,582	3.7	1	.0078	N.S.	.004	.025
	IFNAR1-/-	MHV68	1∶634	1∶358	1∶33,420	1∶309	7.9	82			.018	1.1
	IFNAR1-/-	ISREΔ	1∶708	1∶327	1∶39,948	1∶296	6.6	85	N.S.	N.S.	.017	1
*Key conclusions: M2 ISRE regulates reactivation from splenocytes, but not peritoneal cells, at later times of latency. M2 ISRE does not regulate reactivation in the absence of IFNAR1.*												
28	C57BL6/J	MHV68	1∶1315	1∶1623	∼1∶520,001^6^	1∶31,520	1	1			∼.002	.052
	C57BL6/J	ISREΔ	1∶726	1∶1647	1∶70,013	1∶26,781	∼7.4	1.2	.0068	N.S.	.01	.06
	IFNAR1-/-	MHV68	1∶790	1∶505	1∶19,550	1∶312	∼27	101			.04	1.5
	IFNAR1-/-	ISREΔ	1∶788	1∶511	1∶19,265	1∶323	∼26	99	N.S.	N.S.	.04	1.4
*Key conclusions: M2 ISRE regulates reactivation from splenocytes, but not peritoneal cells, in a second host background. M2 ISRE does not regulate reactivation in the absence of IFNAR1 in the C57BL6/J background.*												
28	IRF2+/+	MHV68	1∶589	1∶472	∼1∶464,159^6^	1∶42,332	1	1			∼.001	.01
	IRF2+/+	ISREΔ	1∶230	1∶387	1∶69,000	1∶42,501	∼6.7	1	.009	N.S.	.003	.009
	IRF2-/-	MHV68	1∶262	1∶315	1∶65,005	1∶42,007	∼7.1	1			.004	.007
	IRF2-/-	ISREΔ	1∶293	1∶330	1∶68,199	1∶41,235	∼6.8	1	N.S.	N.S.	.004	.008
*Key conclusion: M2 ISRE does not regulate reactivation in the absence of IRF2.*												

1 Data for IFNAR1-/- mice on either 129S2 or C57BL6/J backgrounds are shown grouped with the relevant wildtype mice for comparison. Data for IRF2-/- mice are shown grouped with IRF2+/+ littermates for comparison.

2 Calculated by Poisson distribution from limiting dilution reactivation or viral genome PCR assays as described in Methods.

3 Fold increase in reactivation is the ratio of reactivating cells of indicated mouse genotypes to reactivation observed in wildtype mice of the same genetic background infected with MHV68. Note that this ratio is not corrected for differences in viral genome frequency.

4 P value is the value for statistical significance obtained comparing unmanipulated limiting dilution reactivation data sets (Figure 7) of ISREΔ to MHV68 in the same host genotype and at the same time post infection, as determined by Wilcoxon-matched pairs. N.S., not significant (p>0.05).

5 Efficiency of reactivation is defined as the ratio of reactivating cell to latent cells. Note that this ratio is corrected for differences in viral genome frequency. An efficiency of one indicates that all latently-infected cells underwent reactivation. Efficiencies of greater than one indicate the maximal error inherent in detecting single viral genome positive cells as described in Methods.

6 Where reactivation efficiency did not reach 63%, the frequency of cells reactivating was extrapolated from reactivation data sets assuming a sigmoidal dose-response curve with a Hill slope of 1.

### M2 ISRE is not required for MHV68 replication or IFNαβ sensitivity *in vitro*

**Figure 3:**
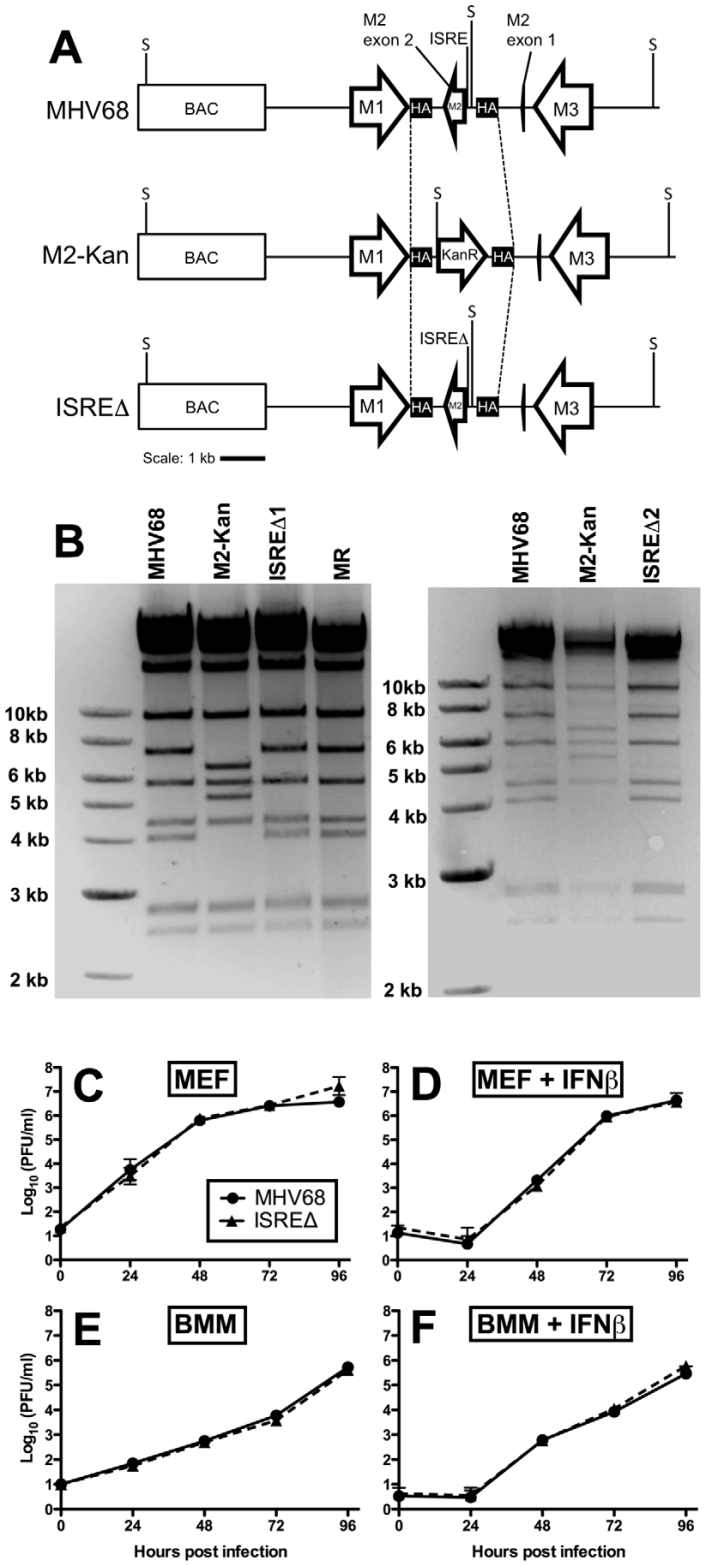
Generation and characterization of MHV68 lacking the M2 ISRE (ISREΔ). **(A)** BAC-mediated mutagenesis was used to generate MHV68-ISREΔ virus. A portion of the M2 intron and the entire M2 second exon were replaced with a kanamycin-resistance cassette (M2-Kan) to permit facile identification of recombinants incorporating the M2 ISREΔ (ISREΔ) mutation in a second round of recombination. KanR, kanamycin resistance cassette. M1, M2, and M3 are MHV68 genes. BAC, bacterial artificial chromosome vector sequences. SpeI restriction sites (S) are indicated. SpeI digest of MHV68, MR, and ISREΔ are predicted to yield fragments of 7.2 and 4 kb in the mutagenized locus, while M2-Kan yields fragments of 6.4 and 5.2 kb. Dashed line indicates the maximum possible regions exchanged during homologous recombination between M2-flanking homology arms (HA) **(B)** Characterization of two independent ISREΔ mutants (ISREΔ1 and ISREΔ2) and a genetically restored marker rescue (MR) construct derived from ISREΔ1. Purified BAC DNA was digested with SpeI and resolved by agarose gel electrophoresis. **(C–F)** Murine embryonic fibroblasts (MEFs, **(C, D)**) or bone marrow-derived macrophages (BMMs, **(E, F)**) from 129S2 mice were either untreated **(C, E)** or treated with 500 U/ml IFNβ **(D, F)** for 18 hours prior to infection with MHV68 or ISREΔ1 at a multiplicity of infection of 0.1 PFU per cell. At the indicated time post infection, cultures were disrupted by freezing and thawing and infectious virus quantified by plaque assay. Shown are mean (+/- SEM) of three independent experiments for each group.

We generated two independent mutant viruses lacking the IRF contact residues in M2 ISRE (ISREΔ1 and ISREΔ2) and a repaired marker rescue (MR) virus (Figure 3A,B). ISREΔ1 replication was identical to MHV68 in murine embryonic fibroblasts (MEFs) (Figure 3C,D) or bone marrow-derived macrophages (BMM) (Figure 3E,F), and ISREΔ1 replication was inhibited normally by pretreatment of cells with IFNβ (Figure 3D,F). Thus, the M2 ISRE is not required for viral replication or inhibition by IFNβ *in vitro*.

### The M2 ISRE represses MHV68 replication at late time points of acute infection in wildtype but not IFNAR1-/- mice

**Figure 4:**
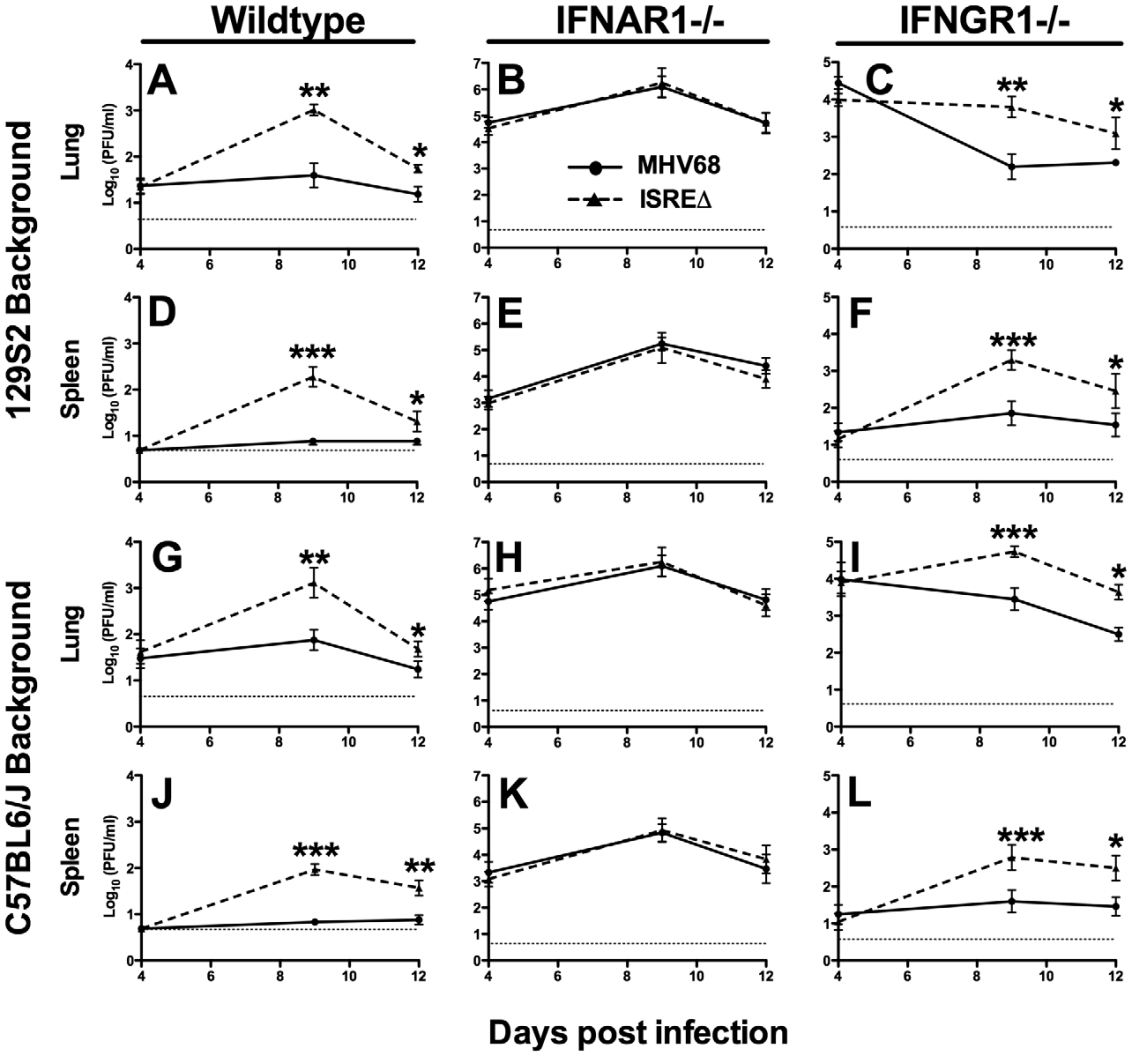
The M2 ISRE represses lytic replication *in vivo* via a mechanism that requires IFNAR1. Mice of the indicated genotype on either a 129S2 **(A–F)** or C57BL6/J background **(G–L)** were infected with MHV68 (solid line) or ISREΔ1 (dashed line) and lung and spleen harvested 4, 9, 12, or 21 dpi. Infectious virus was quantified by plaque assay. Shown are mean viral titers (+/- SEM) from three pooled independent experiments with three to five mice per group. Dashed line indicates the limit of detection. See Figure S2 for control experiments with ISREΔ2 and MR virus demonstrating that increased replication is specific for the ISREΔ mutation. *p≤0.05, **p≤0.01, ***p≤0.001, ****p≤0.0001 by paired, two-tailed t-test comparing ISREΔ to MHV68 in the same host strain at the same time point. Where not indicated, p>0.05.

To determine whether the M2 ISRE regulates acute infection *in vivo*, we infected mice with MHV68 and ISREΔ1 and quantified viral titer in lung and spleen (Figure 4). At 4 dpi, replication of MHV68 and ISREΔ1 in lungs of wildtype mice of two genetic backgrounds was identical, indicating that the M2 ISRE is not required for early acute infection. In contrast, at 9 dpi, we observed a 20- to 30-fold increase of ISREΔ1 replication in both lung and spleen (Figure 4A,D,G,J). Increased replication of ISREΔ1 persisted at 12 dpi (3- to 7-fold upregulated), but no infectious virus of either strain was detectable in spleen at 16, 21, or 28 dpi, indicating that clearance of ISREΔ1 acute infection is not delayed (See Methods). Increased replication of ISREΔ1 was specific for the ISREΔ mutation, since it was observed during infection with ISREΔ2 and was restored to MHV68 levels in MR virus infection (Figure S2). Thus the M2 ISRE represses viral replication at late times of acute infection, suggesting that deletion of M2 ISRE allows MHV68 to bypass some component of the host response. To test whether the host control mechanism uncovered by M2 ISRE deletion requires IFNαβ, we compared MHV68 and ISREΔ1 replication in IFNAR1-/- mice. Replication of both MHV68 and ISREΔ1 was significantly upregulated in IFNAR1-/- mice compared to wildtype mice, but in the absence of IFNAR1 no difference in replication of MHV68 and ISREΔ1 was observed (Figure 4B,E,H,K). As a control for specificity of the IFNAR1 signaling pathway, we infected mice lacking the IFNγ receptor (IFNGR1-/-). These mice displayed increased early replication (4 dpi), but no difference was observed between MHV68 and ISREΔ1 at this time point. However, as observed in wildtype mice, replication of ISREΔ1 was increased 15- to 30-fold at 9 dpi in lung and spleen of IFNGR1-/- mice, and remained elevated 6- to 17-fold at 12 dpi (Figure 4C,F,I,L). Thus, the M2 ISRE functions as a repressor of MHV68 replication at late times of acute infection, and acts by a mechanism that seems to require functional IFNαβ, but not IFNγ, signaling. However, it is possible that the high level of replication of both viruses in IFNAR1-/- mice may obscure the contribution of the M2 ISRE to replication.

### Repression of acute MHV68 replication by M2 ISRE requires IRF2 and B cells

**Figure 5:**
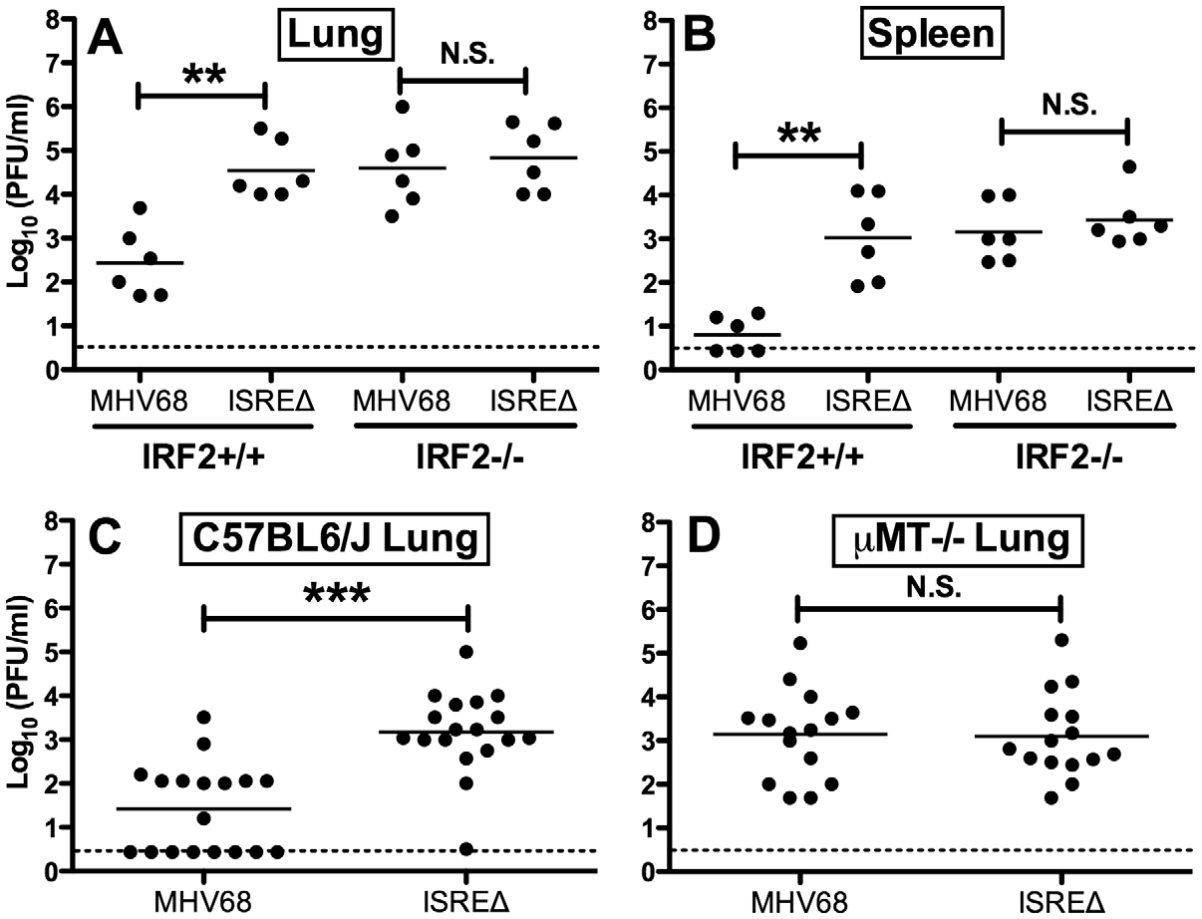
Increased replication of ISREΔ requires IRF2 and B cells. Mice of the indicated genotype were infected with MHV68 or ISREΔ1 intranasally, indicated organs harvested 9 dpi, and infectious virus quantified by plaque assay. Titers present in lung (**A**) or spleen (**B**) of IRF2+/+ or IRF2-/- littermates are shown from two independent experiments. Titers present in lung of C57BL6/J **(C)** or B cell deficient (μMT-/-) **(D)** mice (on a C57BL6/J background) are shown from three to six independent experiments. Dashed line indicates the limit of detection. Shown are mean (bar) and individual viral titers from pooled experiments with three to five mice per group. Dashed line indicates the limit of detection. **p≤01, ***p≤0.001, by paired, two-tailed t-test comparing ISREΔ to MHV68 in the same host strain at the same time point. N.S., not significant (p>0.05).

Since IFNαβ likely controls MHV68 replication by multiple mechanisms, we quantified replication of MHV68 and ISREΔ in IRF2-/- mice as a more specific test of the requirement of IFNAR1-dependent signaling in regulating MHV68 replication via the M2 ISRE. While the replication of ISREΔ1 was increased ∼100-fold relative to MHV68 in IRF2+/+ mice, in IRF2-/- littermates MHV68 replication and lytic gene expression rises precisely to the level of ISREΔ1, and the two viruses are statistically identical (Figures 5A,B). Importantly, titers in IRF2-/- mice are >10-fold lower than the maximum observed in IFNAR1-/- mice (Figure 4B,E,H,K), suggesting that IRF2-independent increases in replication of ISREΔ virus should be evident if they existed. The absence of increased replication of ISREΔ in IRF2-/- mice suggests that the M2 ISRE functions solely in response to IFNαβ-dependent IRF2 to decrease replication. The observation that ISREΔ1 replicates at higher levels than MHV68 during acute infection was unexpected, since M2 is dispensable for acute replication *in vitro* and *in vivo* [9]. All known functions of M2 are B-cell-specific and include inducing B cell entry into and egress from the GC reaction, and triggering B cell differentiation into plasma cells, the predominant cell type supporting viral reactivation *in vivo* [10], [15]. However, latently-infected B cells are detectable in the lung early during acute MHV68 infection [24]. Thus, we reasoned that increased replication of ISREΔ during late acute infection may be due to premature reactivation in infected B cells. To test this hypothesis, we infected B cell deficient mice (μMT-/-) with MHV68 and ISREΔ1 and quantified replication (Figure 5C,D). MHV68 replication increased approximately 20-fold in µMT-/- mice, which we speculate may be due to redirection of MHV68 virions to a purely lytic infection in the absence of B cells as targets for latency. Alternatively, B cells may exert an indirect antiviral effect on MHV68 replication. However, this effect must still require the M2 ISRE for function since ISREΔ replication is identical in wildtype and µMT-/- mice. ISREΔ1 replication was indistinguishable from MHV68 replication in lungs of µMT-/- mice at 4, 9, and 12 dpi (Figure 5D and data not shown). Neither MHV68 nor ISREΔ1 virus was detected in spleen of µMT-/- mice, consistent with a critical role for B cells in spread ([25] and data not shown). Thus, the increased replication phenotype of ISREΔ1 requires B cells, suggesting that newly infected B cells require IFNAR1- and IRF2-dependent repression of M2 expression to prevent premature viral reactivation.

### M2 ISRE specifically represses M2 expression during latency via an IRF2-dependent mechanism

**Figure 6:**
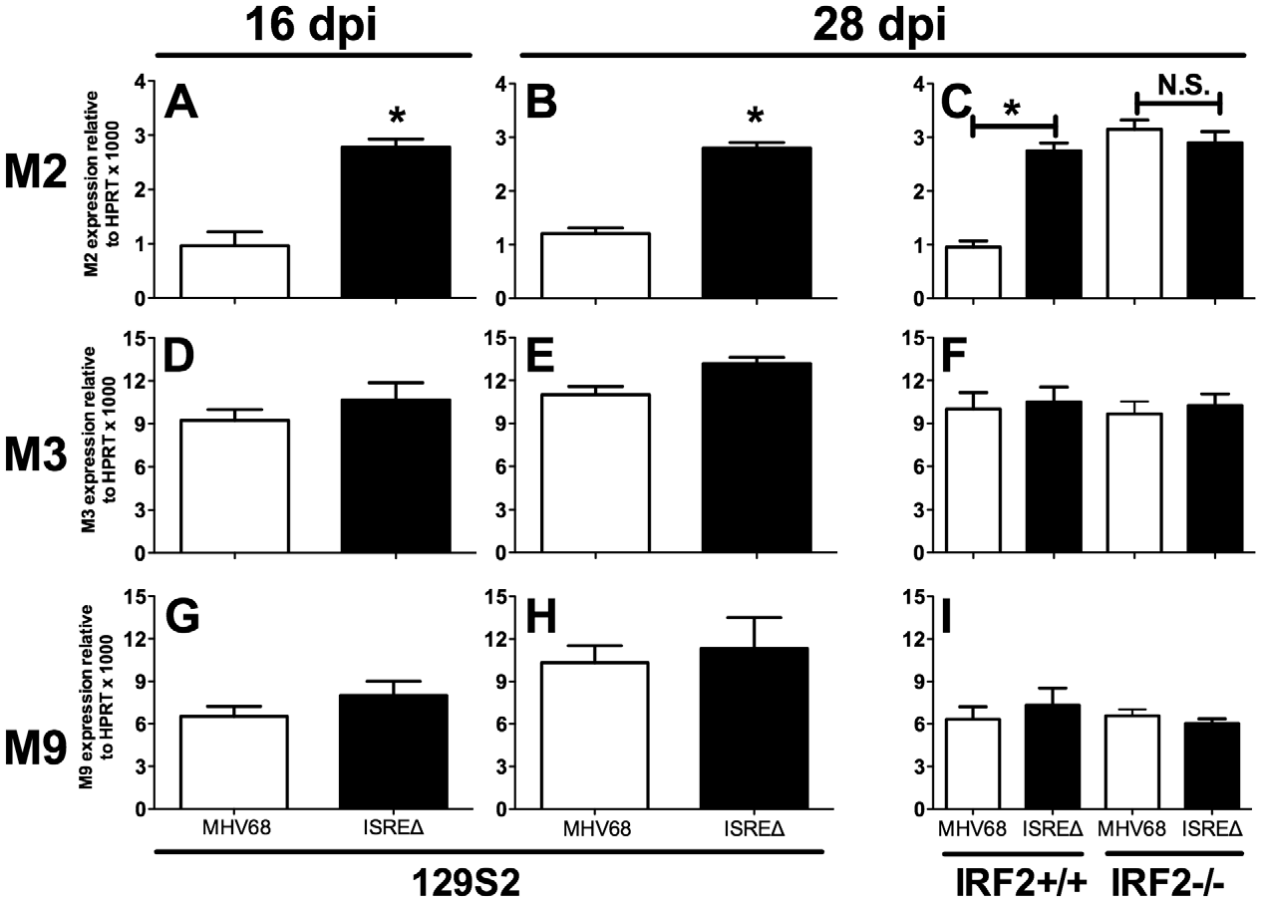
The M2 ISRE requires IRF2 to represses M2 transcript levels during latency *in vivo*. Total RNA was harvested from splenocytes of 129S2 or IRF2+/+ and IRF2-/- littermate mice. Viral RNAs encoding M2 (**A–C**), M3 (**D–F**), or M9 (**G–I**) were detected using quantitative RT-PCR. Viral gene expression was normalized to cellular mRNA standard (HPRT) and subsequently to viral genome copy number (due to two-fold increases in latently-infected cells present in ISREΔ infection at 28 dpi [Table 1]) and is expressed as fold increase relative to MHV68 infection in wildtype mice of the appropriate genetic background. Shown are mean (+/- SEM) from three pooled independent experiments with three to five mice per group. *p≤0.05 by paired, two-tailed t-test, comparing ISREΔ to MHV68 in the same host strain at the same time point. Where not indicated, p>0.05. This phenotype was confirmed using the ISREΔ2 virus (two independent experiments, data not shown). See Figure S3 that compares the kinetics of M2, IFNβ, and IRF2 expression in spleen of infected mice.

To determine if the M2 ISRE regulates M2 expression during latency, we quantified M2 mRNA in spleens at times when lytic replication is absent (Figure 6). M2 mRNA was significantly upregulated at both early (16 dpi) and later (28 dpi) times during latent ISREΔ1 infection (Figure 6A–C). Upregulation was specific for M2 mRNA and was not observed for viral M3 or M9 transcripts (Figure 6D–I). Importantly, in IRF2-/- mice M2 transcript expressed by MHV68 increased precisely to the level observed in ISREΔ1 infection, while M3 and M9 expression efficiency were unaltered. Thus, M2 expression is specifically repressed during latency by an M2 ISRE- and IRF2-dependent mechanism. When we compared the kinetics of IFNβ, IRF2, and M2 expression in the spleen, we detected elevated IFNβ and IRF2 mRNA in spleen by 4 dpi (Figure S3). At this timepoint no M2 mRNA is detectable, likely because virus has not yet reached the spleen (Figure 4). From 9–28 dpi with MHV68, M2 mRNA is present in the spleen at a low but constant level. In contrast, M2 expression is upregulated ∼3–4-fold at all time points in ISREΔ infection, indicating that the M2 ISRE reduces, but does not completely silence, M2 expression (Figure S3). Thus, our data indicate that M2 expression is controlled by at least two promoter elements: a 5′ promoter proximal to the transcription start site [20] and the intronic ISRE we report here (Figure 1).

### M2 ISRE represses reactivation from splenic cells but not peritoneal cells

**Figure 7:**
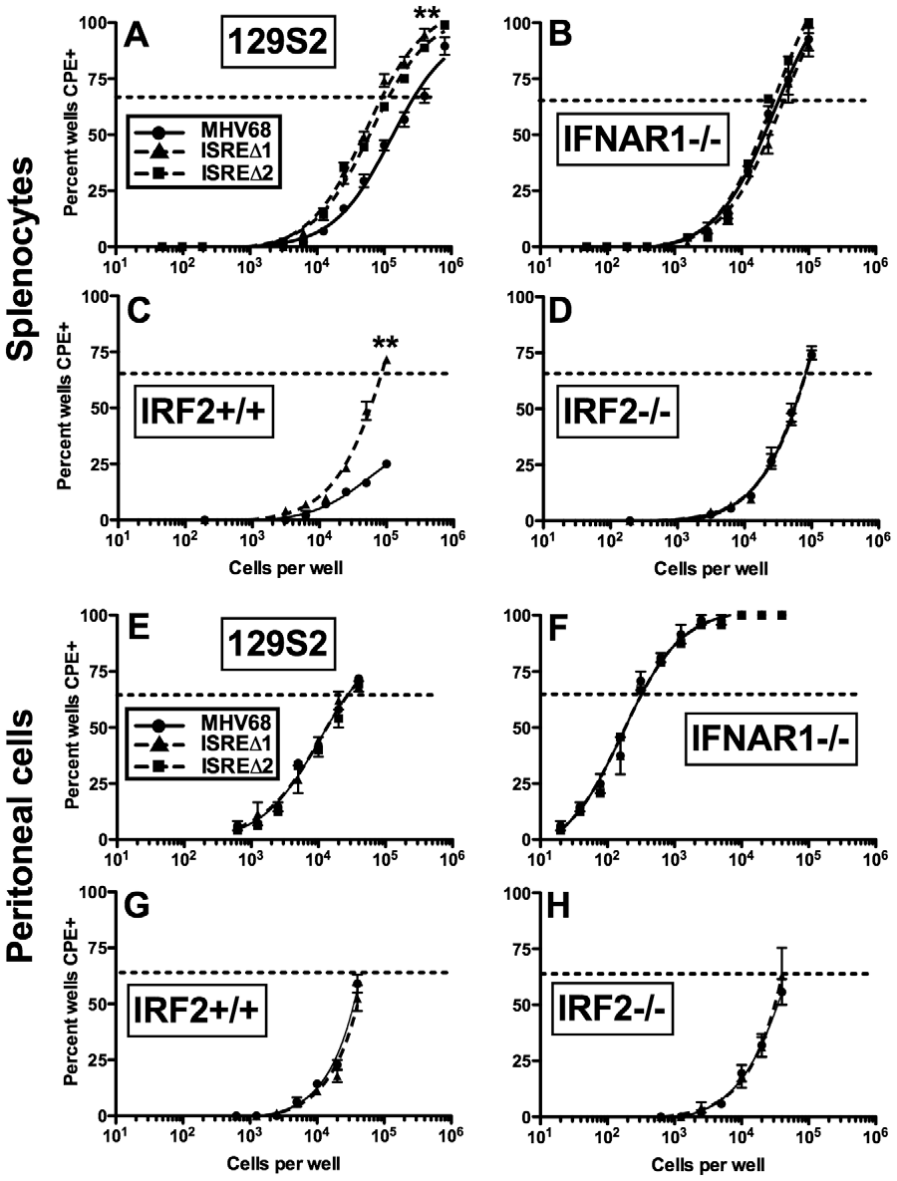
The M2 ISRE requires IFNAR1 and IRF2 to represses viral reactivation from latent splenocytes. Splenocytes (**A–D**) and peritoneal cells (**E–H**) were harvested from 129S2, IFNAR1-/- (129S2 background), IRF2+/+, or IRF2-/- littermate mice latently-infected (28–35 dpi) with MHV68, ISREΔ1 or ISREΔ2. Viral reactivation was quantified using an *ex vivo* limiting dilution reactivation assay. Shown is mean (+/- SEM) viral reactivation from at least three independent experiments with three to five mice per group. See Figure S4 for limiting dilution PCR analysis of viral genome frequencies present in these samples. Dashed line indicates the point of 63% reactivation-positive wells as determined by non-linear regression, which was used to calculate the frequency of cells reactivating (Table 1). Note that additional, higher dilutions of cells were plated in **A** to permit direct interpolation of reactivation frequency of MHV68 in wildtype mice. Reactivation frequency of MHV68 in IRF2+/+ mice was extrapolated as described in Table 1. **p≤0.01 by Wilcoxon matched pairs test comparing ISREΔ to MHV68 in the same host strain. Similar results were obtained in wildtype and IFNAR1-/- mice on a C57BL6/J background (Table 1).

M2 overexpression is sufficient to drive viral reactivation from plasma B cells [15]. To determine whether repression of M2 by IRF2 decreases MHV68 reactivation, we performed *ex vivo* reactivation assays with splenocytes and peritoneal exudate cells (PECs) from mice infected with MHV68, ISREΔ1, or ISREΔ2 (Figure 7, Figure S4, Table 1). Splenocytes from wildtype mice infected with either ISREΔ1 or ISREΔ2 showed a significant four-fold increase in reactivation compared to MHV68 at both 16 and 28 dpi (Table 1 and Figure 7A,C). At 16 dpi, increased reactivation was solely attributable to increased reactivation efficiency, since the frequency of latently-infected cells was equivalent (∼1∶1400) in mice infected with either virus. However, at later times, increased reactivation during ISREΔ infection was a composite effect of both increased numbers of latently-infected cells (Figure S4) and increased efficiency of reactivation (Table 1). Increased reactivation efficiency of ISREΔ required IFNAR1 and IRF2, since in IFNAR1-/- and IRF2-/- mice MHV68 and ISREΔ mutant viruses reactivated with identical frequencies that are increased relative to those observed in MHV68-infected wildtype mice (Figure 7B–D, Table 1). Importantly, in this assay, <10% of latently-infected cells reactivate (Table 1), permitting sufficient upward dynamic range for IFNAR1-independent or IRF2-independent effects of the M2 ISRE to be observable if they existed. Increased reactivation of ISREΔ is likely B cell-specific, since reactivation of MHV68, ISREΔ1, and ISREΔ2 from PECs was identical under all conditions (Table 1 and Figure 7E–H). The major latent cell in the spleen is the B cell, while in PECs most latent virus resides in macrophages [26]. Taken together, these genetic data strongly suggest that MHV68 reactivation from B cells is repressed by IFNαβ-driven, IRF2-mediated repression of M2 expression, acting through the M2 ISRE.

#### M2 ISRE attenuates MHV68 virulence

**Figure 8:**
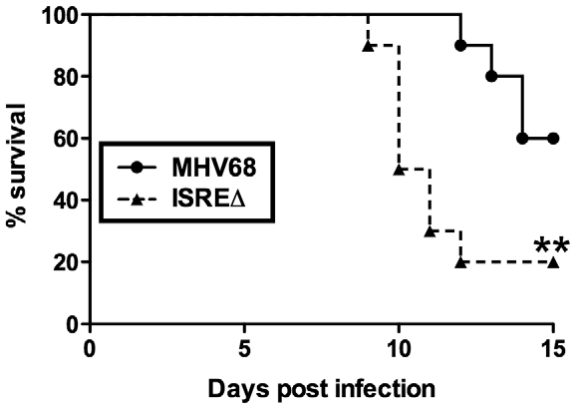
The M2 ISRE attenuates MHV68 virulence. IFNγ-/- mice on a BALB/c background were infected intranasally with 4×10^5^ PFU of MHV68 or ISREΔ1 and monitored for lethality daily. Shown is a result from two independent experiments with five mice per experimental group. Survival curve was generated using Kaplan-Meier analysis and statistical significance (**p≤0.01) was calculated using Log-Rank test.

The increased replication and reactivation of ISREΔ relative to MHV68 suggested that the M2 ISRE may function to minimize host pathology during acute infection. To test this hypothesis, we infected IFNγ-/- mice on a BALB/c background, which succumb to acute lethal MHV68 pneumonia [27]. Following intranasal infection, ISREΔ-infected IFNγ-/- mice showed significantly increased lethality compared to mice infected with MHV68 (Figure 8). Thus, the M2 ISRE functions to attenuate acute MHV68 infection, likely by acting to reduce viral replication in response to IFNαβ-induced IRF2.

## Discussion

**Figure 9:**
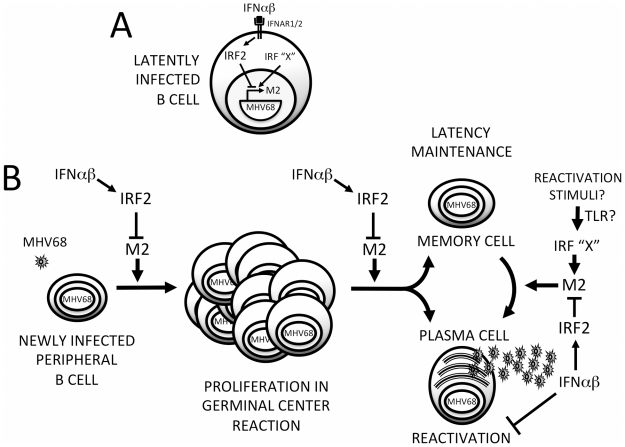
Model for M2 ISRE regulation of MHV68 infection *in vivo*. **(A)** Following induction of IFNαβ expression during MHV68 infection, IFNAR1-dependent signaling upregulates IRF2, which binds to the M2 ISRE and represses M2 transcription. Other IRFs (IRF “X”) may serve as positive or negative regulators of M2 expression during different stages of the viral life cycle. Specifically, activation of transcriptional activator IRFs downstream of TLRs may regulate MHV68 reactivation. **(B)** IRF2-dependent M2 repression regulates the efficiency of latently-infected B cell entry into the GC reaction, exit of latent cells from the GC, and differentiation into plasma B cells that can support reactivation. Repression of M2 expression by IFNαβ-dependent IRF2 expression may provide a crucial mechanism for controlling proliferation and differentiation of latently-infected B cells and timing viral reactivation to periods of localized immune quiescence. Additional IFNαβ-dependent but M2 ISRE/IRF2-independent mechanisms exist for regulating MHV68 reactivation from both B cells and macrophages.

We have uncovered a previously unappreciated mode of interaction between viruses and IFNαβ: rather than evading this antiviral system, MHV68 directly cooperates with it to silence replication during establishment of latency. This strategy relies on IFNαβ-induced IRF2 to regulate critical cell differentiation decisions following viral infection of B cells (Figure 9). Shortly after B cell infection, the M2 ISRE is either unoccupied or may be bound by transactivating IRFs (IRF”X”, Figure 9A). M2 expressed at this time drives B cells into a GC reaction, resulting in expansion of latently-infected memory and plasma cells. As replication peaks, IFNαβ induces IRF2, which binds the M2 ISRE and represses M2 transcription. M2 silencing would decrease entry of infected B cells into the GC (Figure 9B), reducing overall latent load [10]. M2 is sufficient to promote differentiation into plasma cells [15] and the majority of viral reactivation is derived from plasma cells [15]. Thus, IRF2-dependent M2 repression is also expected to decrease reactivation. When we perturb this regulatory switch using either IRF2-/- mice or ISREΔ virus, the result is a substantial increase in viral replication during late acute infection (Figure 4), which we attribute to premature reactivation from newly infected B cells (Figure 5) driven toward plasma cell differentiation by overexpression of M2 (Figure 6). This increase in reactivation is still evident during ISREΔ latency, and results in increased latent load over time (Figure 7). Additional IFNαβ-dependent mechanisms exist to control MHV68 replication and reactivation, since the M2 ISREΔ mutation does not fully recapitulate the dysregulation of these processes observed in IFNAR1-/- mice [5]. While the simplest mechanism that is consistent with our genetic and biochemical data involves IFNαβ-induced IRF2 binding to the M2 ISRE to reduce M2 expression in infected B cells during latency expansion in the spleen, other interpretations are possible. For example, it is also conceivable that B cells and IRF2 exert M2 ISRE-dependent control of viral replication and reactivation in a *trans-*acting manner, rather than directly in the infected B cell.

We propose the term “cooperative subversion” to describe this regulatory approach. The cooperative nature of the strategy is evident by the lack of IFNAR1-dependent inhibition of ISREΔ replication we observe late in acute infection (Figure 4), which correlates with increased lethality in a moderately immune compromised (IFNγ-/-) host (Figure 8). This indicates that a primary function of the M2 ISRE is to reduce viral replication in cooperation with the host IFNAR1 signaling pathway. To our knowledge this is the first element identified in a herpesvirus genome that directly engages a host ISG to decrease viral replication. We hypothesize that this mechanism provides a developmental switch to MHV68: once it has established a latent load that assures life-long persistence, viral gene expression shifts to a pattern that will prevent host pathology and lethality, minimize the risk of B cell transformation, and reduce viral antigen presentation. This also provides a potential strategy for γHVs to target reactivation to periods of localized immune quiescence during long-term latency, by permitting high level M2 expression only in microenvironments where IFNαβ secretion has decreased, when replication is more likely to be productive. Thus, we believe this mechanism is simultaneously cooperative and subversive, but must be contrasted with overt IFNαβ evasion strategies that are well documented during lytic infection with many viruses [8].

The γHVs are uniquely suited to cooperation with the host immune response. They need minimal replication to establish latency, and instead rely on virus-driven proliferation of B cells to seed the host. Indeed, the frequency of MHV68 latency is independent of inoculum dose [28], and replication-defective MHV68 can establish latent infection [24], [29]. γHVs can rely on lifelong transmission to spread to a new host, obviating the need for high-level persistent replication; asymptomatic reactivation at mucosal surfaces is instead the rule [30]. Since γHVs rely on the health of the host to promote spread, it is likely a selective advantage for them to cooperate with the host immune response to prevent unbridled amplification of latent B cells and predisposition of the host to neoplasia. Consistent with this hypothesis, there is evidence that the dominant T cell epitopes of EBV are selected for conservation, rather than evasion [31]. Our data demonstrate that cooperation with innate antiviral cytokines may also function during acute and latent infection. Whether this attenuating, cooperative effect of the M2 ISRE is the primary function selected during virus-host coevolution is not directly discernable from our studies.

We found unexpectedly high-level IFNβ expression during latency, at times where we detect no infectious virus (Figure 2). In subsequent studies, we found IFNβ-producing cells in the spleen of latent mice at 48 dpi, and significant upregulation of ISGs at 90 dpi (data not shown, manuscript in preparation). It is not clear what viral triggers or host sensors lead to this sustained IFN production. However, these data demonstrate that the IFNαβ-driven response is not limited to acute infection, but extends well into latency. Our data also indicate that host genes may not directly mediate all “antiviral” effects of IFNαβ, but that viral elements may be required to repress replication in response to IFNαβ. By 28 dpi, nearly half of the antiviral effect of IFNαβ in the spleen is mediated by the M2 ISRE, since MHV68 reactivation in IFNAR1-/- mice is upregulated 7.9-fold yet deletion of the M2 ISRE alone upregulates reactivation 3.7-fold (Table 1, 129S2 background, 28 dpi). Interestingly, the M2 ISRE has no apparent function during latency in peritoneal macrophages, a cell type where latency and reactivation are also independent of M2 (Figure 7) [9]. This confirms that the ISREΔ mutation does not dysregulate latency and reactivation in all cell types, and indicates that IFNAR1-dependent pathways active in latent macrophages remain to be defined.

Our data indicate a novel, direct antiviral function for IRF2 during γHV latency. Although IRF2-/- mice have immune defects that disrupt control of some acute viral infections [7], [32]–[34], they clear MHV68 replication, establish latency at frequencies nearly identical to IRF2+/+ littermates, and regulate viral reactivation from the peritoneal compartment normally. This indicates that the immune modulatory functions of IRF2 are not essential for an effective response to MHV68 infection. In IRF2+/+ littermates, ISREΔ virus replicates to 100-fold higher titer in the lung, expresses significantly more M2 mRNA, and reactivates with enhanced efficiency relative to MHV68. Importantly, we found that MHV68 replication, M2 expression, and reactivation rises precisely to that of ISREΔ in IRF2-/- mice (Figures 5,6,7, Table 1). Thus, the M2 ISRE can only repress M2 expression and restrain MHV68 replication and reactivation in a host that expresses IRF2. This confirms that these phenotypes are not the result of generalized immune defects during latency in IRF2-/- mice, but almost certainly require the interaction between IRF2 and the M2 ISRE observed in Figure 1. Since IRF2 has oncogenic properties [35], our data raise the question of whether γHV-induced IRF2 may play a tumor-promoting role during γHV latency.

Our data indicate that M2 expression is regulated by two distinct promoter elements. The first identified M2 promoter is located 5′ to the M2 transcription initiation site, is functional in murine B cells, and binds undefined transcription factors [20]. Our data indicate that the M2 ISRE can decrease the firing rate of this 5′ promoter when it is occupied by IRF2. IRF2 generally functions as a transcriptional repressor, but its function is modulated by several posttranslational modifications, including proteolysis (which exposes a transcriptional transactivation domain) [36], sumoylation [37], acetylation [38], phosphorylation [39], and interaction with other IRFs and co-factors [40]. The modification state of IRF2 and levels of other IRFs that compete for binding to the M2 promoter is likely to be dynamically regulated. Importantly, IRF2 binding to the M2 ISRE does not completely silence the M2 locus, thereby allowing expression of reduced M2 levels in the face of the host IFNαβ response. We speculate that during establishment and early expansion of latency, IRF2-mediated reduction in M2 expression is required to prevent untimely viral reactivation, which can be triggered directly by M2 overexpression [15].

A spatiotemporally regulated balance of IRFs likely determines the expression of M2 at distinct stages of the MHV68 latent life cycle. It is noteworthy that multiple intrinsic and extrinsic stimuli that induce γHV reactivation activate IRFs. Toll-like receptor (TLR) stimulation with multiple viral and bacterial molecules triggers reactivation of KSHV and MHV68 [41], [42]. IRFs that are activated downstream of TLR stimulation include IRFs 1, 3, 5, and 7 [43], [44]. In addition, DNA damage both activates IRF5 and induces MHV68 reactivation [45], [46]. These data suggest that TLR- or stress-induced IRF activation may serve to displace IRF2 from the M2 ISRE, inducing M2 expression and reactivation. However, there is no evidence for a transactivator bound to the M2 ISRE at the time points we assess, since M2 transcript levels are upregulated to the same extent when either the ISRE or IRF2 is deleted (Figure 6). It has been reported that overexpression of M2 may impair IFNαβ-induced signaling pathways [47]. Although this function has not been confirmed in lymphocytes expressing physiologic levels of M2, this strategy may enable sustained M2 expression once reactivation is induced, by acting as a negative feedback loop to prevent IFNαβ-driven IRF2 expression and silencing of M2 transcription during latency establishment or reactivation.

Complex but poorly understood relationships exist between the human γHVs and IRFs. KSHV encodes viral IRF homologs (vIRFs) that modulate function of host IRFs [48], [49]. vIRF3 is expressed during KSHV latency, when it antagonizes IRF5 and p53 and enhances transactivation by IRFs 3 and 7 [49], [50], and is required for latent cell proliferation and survival [51]. EBV interacts with numerous IRFs to regulate latent promoters. IRF2 binds to the EBNA-1 Qp promoter during restricted latency programs I and II [18], [52]. Although IRF2 represses Qp in some EBV-infected B cell lines [18], other reports indicate that it can upregulate Qp-driven EBNA-1 expression [52], [53]. In addition, the promoter of EBV LMP1, a viral CD40 signaling mimic, contains an ISRE and is induced by IRF7 and repressed by IRF5 (Table 2). The consequences of EBV and KSHV promoter-IRF interactions for the infected cell *in vivo* are unknown.

**Table 2:** Gammaherpesvirus ISREs regulating genes involved in lymphocyte latency and immortalization.

Host	Virus	ISRE^1^	Position (Accession)	Genes regulated^2^	IRFs bound	Function	References
Wood mice *(Apodemus sylvaticus)*	MHV68	GAAAACGAAACC	4612–4623 (U97553.2)	M2	IRF2	Suppresses M2 expression; reduces replication, reactivation, and virulence	This report
Wood mice *(Apodemus sylvaticus)*	Wood mouse herpesvirus (WMHV, strain WM8)	GAAAACGAAACC	4520–4531 (GQ169129.1)	5′ to M2 homolog	?	?	ISRE not previously reported [54]
White-toothed shrew *(Crocidura russula)*	Brest herpesvirus (BRHV, An711)	GAAAACGAAACC	5996–6007 (EF495130.1)	5′ to M2 homolog	?	?	ISRE not previously reported [54]
Human	EBV	GAAAACGAAAGT	50118–50129 (NC_007605.1)	EBNA1	IRF1, IRF2, IRF5, IRF7	Regulates EBNA1 Qp promoter utilization	[18], [52], [53], [60]
Human	EBV	GAAATGGAAAGG	169301–169314 (NC_007605.1)	LMP1	IRF5, IRF7	Regulates LMP1 expression level	[17], [61]
Human	KSHV (strain GK18)	GAAAACGAAAGC	137746–137735 (AF148805.2)	5′ to K1	?	?	ISRE not previously reported
Squirrel monkey (Saimiri sciureus)	Herpesvirus saimiri (HVS)	GAAAGTGAAACT	2710–2699 (X64346)	5′ to STP-A11	?	?	ISRE not previously reported

1 Nucleotide sequences for the indicated accessions were searched for the consensus ISRE sequence GAAANNGAAA [21]. Shown are ISREs located 5′ to the major lymphocyte immortalization latency genes of each virus.

2 Proximal latency-associated genes are noted. Where regulatory function of the ISRE has been experimentally confirmed, IRFs bound and regulated viral genes and references are indicated in adjacent columns.

Several of the IRFs implicated in regulating KSHV and EBV genes (including IRFs 2, 5, and 7) are induced by IFNαβ [21]. Little attention has been given to the possibility that IRF-induced or -repressed EBV and KSHV latent gene expression may be responsive to the inflammatory environment of the infected cell. We demonstrate that a latent gene controlling B cell differentiation and reactivation is repressed by IFNαβ. Interestingly, LMP1 and M2 are located in homologous regions of the viral genome [3], and our work here demonstrates that like LMP1, M2 is regulated by a conserved ISRE. While the importance of IRF-mediated LMP1 regulation *in vivo* is unknown, our data suggest that EBV-infected cells may utilize this circuit to fine-tune the balance between latent B cell proliferation and reactivation in response to host inflammation. Intriguingly, we find consensus ISREs in the 5′ regions of the major lymphocyte immortalization genes of EBV, KSHV, the primate γHV herpesvirus saimiri, and two newly sequenced rodent γHVs [54], suggesting that subversion of host IRFs to regulate the switch between lytic and latent infection is an evolutionarily ancient invention (Table 2). Future studies with MHV68 will enable dissection of the dynamics of viral promoter/IRF interactions that may permit rational intervention to manipulate the balance between viral latency, reactivation, and oncogenesis.

## Methods

### Ethics statement

This study was carried out in strict accordance with the recommendations in the Guide for the Care and Use of Laboratory Animals of the National Institutes of Health. Mice were handled according to all applicable institutional, state, and federal animal care guidelines, under animal care protocols approved by the Purdue University (animal welfare assurance #A3231-01, protocol #06-115) and Wake Forest University Animal Care and Use Committees (animal welfare assurance #A3391-01, protocol #A11-007). Veterinary technicians or laboratory staff assessed animal health at least once daily. Moribund mice were humanely euthanized.

### Cell culture, reagents, viruses and quantitation of viral growth

All cells were maintained in DMEM containing 10% fetal bovine serum (DMEM/10). MEFs were harvested from embryonic d15–18 129S2 and C57BL6/J mice. Bone marrow was harvested from the femur of 129S2 mice and differentiated *in vitro* to produce BMM [55]. Bone marrow was cultured for four days on 100 mm polystyrene dishes in 10 ml endotoxin-free DMEM/10 supplemented to contain 20% vol/vol L929 cell supernatant, 5% vol/vol horse serum, 2 mM L-glutamine, and 1 mM sodium pyruvate. On day four of differentiation, 10 ml of endotoxin-free DMEM/10 supplemented to contain 10% vol/vol L929 cell supernatant, 5% vol/vol horse serum, 2 mM L-glutamine, and 1 mM sodium pyruvate was added to each plate. On day seven of differentiation, cells were detached from dishes using PBS (Ca^2+^/Mg^2+^-free, 1 mM EDTA) and scraping. Wildtype and recombinant virus stocks were generated from wildtype MHV68 propagated as a bacterial artificial chromosome (BAC) [56]. To generate infectious virus stocks, column-purified BAC DNA was transfected into BALB/3T12 cells (ATCC CCL-164) stably transduced with cre recombinase to permit deletion of bacterial sequences. Viruses were passaged at low multiplicity of infection for three generations in BALB/3T12-cre cells prior to use. Organs were disrupted with 1 mm silica beads using a Minibeadbeater 16 (Biospec Products) in 1 ml DMEM/10. Viral titers were determined by plaque assay on BALB/3T12 monolayers [5].

### Viral growth curves

For *in vitro* growth curves, 6×10^4^ MEFs or BMM were plated in 48 well tissue culture treated plates. Immediately after plating, MEFS were incubated with 500 U/ml rIFNβ (PBL laboratories) overnight (∼18 hours) prior to infection. BMMS were kept at 37°C for 24 hours prior to overnight treatment with IFNβ. Cells were infected with 0.1 plaque-forming units (PFU) MHV68 or ISREΔ per cell in an inoculum volume of 0.1 ml DMEM/10 for one hour at 37°C. Inocula were aspirated, cells were washed twice with 37°C PBS, and incubated in DMEM/10. Plates were frozen at −80°C at indicated time points. Plates were frozen and thawed twice prior to plaque assay.

### BAC mutagenesis and generation of recombinant viruses

Recombinant viruses were generated using BAC mediated mutagenesis as described [56]. The M2 locus (nt. 3791–4700) relative to Genbank Accession U97553.2, [3] was replaced with a kanamycin resistance cassette to generate M2-Kan BAC. Two M2 homology arms (5′ arm: nt 3301–3790, 3′ arm: nt 4701–5201) were amplified by PCR and cloned on either side of a kanamycin resistance cassette in allelic exchange mating vector pGS284 to generate pGS284/M2/Kan. MHV68 BAC was mated to pGS284/M2/Kan by cross streak on LB agar plates, and the expected genomic configuration of kanamycin-resistant clones (M2-Kan BAC) arising from host strain intersections were confirmed using a minimum of four restriction endonucleases that yield diagnostic fragment lengths. M2-Kan BAC was mated to pGS284 containing the entire M2 locus (generated by PCR using 5′ homology arm sense and 3′ homology arm antisense primers) that was mutagenized via PCR to encode the M2 ISREΔ mutations as indicated in Figure 1 (pGS284/M2/ISREΔ). Kanamycin sensitive recombinants were identified by replica plating and expected genomic configuration confirmed by restriction digest. Two independent ISREΔ mutant clones were generated using independent stocks of wildtype MHV68 BAC. Mating of ISREΔ1 to pGS284/M2/Kan and subsequent replacement of the M2 locus by mating to pGS284 containing the wildtype M2 sequence generated a genetically repaired marker rescue virus, M2-MR. All PCR amplified homology arms and mutagenized sequences were confirmed by DNA sequencing over the entire length of the construct, and resulting mutant viral BAC DNA was directly sequenced to confirm incorporation or repair of mutations. To generate infectious virus stocks, column-purified BAC DNA (4 µg) was transfected using Fugene HD (Roche) into BALB/3T12 cells that were stably transduced with cre recombinase to permit deletion of the BAC backbone. BAC sequence elimination was confirmed after three passages in BALB/3T12-cre using indirect fluorescence for EGFP expressed from the BAC locus.

### Mice and infections

Age- and sex-matched mice (7-12 weeks of age) were used for all experiments. Wildtype, IFNAR1-/-, and IFNGR-/- mice on 129S2 (old designation, 129/SvPas) background have been described [57]. Wildtype, IFNAR1-/-, IFNGR-/- mice on C57BL6/J background were obtained from Dr. Herbert Virgin (Washington University). Dr. Stephanie Vogel (University of Maryland) donated IRF2-/- mice on C57BL6/J background [58]. B cell deficient (μMT-/-) and IFNγ-/- BALB/c (strain C.129S7(B6)-*Ifng^tm1Ts^*/J) were purchased from Jackson laboratories and are the only genotypes used in this study that were not derived from in-house breeding. Isoflurane-anesthetized mice received 100 PFU intranasally in 40μL of DMEM/10. For viral pneumonia induction (Figure 8), IFNγ-/- BALB/c received 4×10^5^ PFU intranasally. Mice were humanely euthanized in Isoflurane prior to tissue harvest.

### EMSA and ChIP

Nuclear extracts were prepared from splenocytes of latently-infected C57BL6/J mice 28–35 dpi using Pierce NE-PER kit and protein concentration was determined using Bio-Rad RC/DC Kit. For EMSA, probes used were

M2 ISRE: 5′-TTACCTGAAAACGAAACCTCATCA-3′

and M2 ISREΔ: 5′-TTACCTGGAACCTGAACCTCATCA-3′.

^32^P-labeled complementary oligonucleotides were hybridized to generate double stranded (ds) probes. Ds probes were separated from free radiolabeled dUTP by size exclusion chromatography using Sephadex G-50 columns (Roche). Radiolabeled, ds probes were incubated with nuclear extracts and resolved on acrylamide gel [59]. Five µg of protein was incubated in a reaction with 1X binding buffer (40 mM KCl, 20 mM HEPES pH 7.6, 1 mM MgCl_2_, 1 mM EGTA, 0.5 mM DTT), 0.32 mg/ml poly dI-dC (Sigma-Aldrich), 0.02 mg/ml plasmid pgL4.10, 4 mM AMP (Sigma-Aldrich) and 1×10^5^ cpm of radiolabeled probes in a total volume of 12.5 µl at room temperature for 30 minutes. Complexes were resolved on 6% nondenaturing acrylamide-20 mM TBE gel at 4°C. For supershift, two µg of gel-shift certified antisera raised against mouse IRF2 (Santa Cruz #H229 and #C19) were added to gel shift reactions. For competition assays, ^32^P-labeled M2 ISRE probe and nuclear extract were incubated with increasing concentrations of ds unlabeled M2 ISRE or M2 ISREΔ probes. Dried gels were exposed to storage phosphorimager plates and images analyzed using Bio-Rad PDQuest software.

For ChIP, splenocytes (6×10^7^) from latently-infected 129S2 mice were fixed in 1% formaldehyde, washed in PBS, and sheared using a Misonix S3000 Sonicator. Resulting chromatin had an average length of 500–1000 base pairs. Chromatin was incubated overnight with two µg anti-IRF2 (H229, Santa-Cruz Biotech) and immunoprecipitated with protein A/G sepharose. Immunoprecipitated DNA was reverse-crosslinked, phenol/chloroform extracted, ethanol precipitated, and amplified using conditions, PCR primers, and thermal cycling parameters detailed in Supporting Protocol S1. Control (no antibody, or irrelevant rabbit antiserum) immunoprecipitated chromatin yielded no amplicons for any primer set (not shown).

### Quantitation of viral and host mRNA

Total RNA was isolated from intact organs (during lytic infection) or erythrocyte-depleted splenocytes (during latent infection) by silica bead disruption in Trizol (Invitrogen) and subjected to RNA cleanup (Qiagen RNAeasy Kit) and DNAse treatment (Ambion Turbo DNAse Kit). Total RNA (1.5 µg) was used for cDNA synthesis (Invitrogen Superscript Kit) followed by real time PCR on an ABI 7300 using primers for host HPRT, IFNβ, IRF2 or viral M2, M3, or M9 genes. The primers for housekeeping gene HPRT, IFNβ, IRF2 or viral gene M2 span exon-intron junctions, and all amplicons were resolved on agarose gel electrophoresis to confirm predicted size. Amplicons for M2 were sequenced and confirmed that ISREΔ mutations did not alter M2 splicing. M3 and M9 are unspliced viral transcripts; parallel reactions performed in the absence of reverse transcriptase indicated that samples were free from contaminating viral genomic DNA. For quantitation of viral episome number, DNA was harvested from erythrocyte-depleted splenocytes and quantitative PCR analysis performed using primers specific to GAPDH or v-cyclin (ORF72) genomic DNA. Detailed analysis and normalization equations are described in Supporting Protocol S2.

### Limiting dilution assay to quantify frequency of latently-infected and reactivating cells

Frequencies of viral genome positive and reactivating cells were determined as described [5]. Briefly, on the indicated day post infection mice were euthanized and spleen and peritoneal exudate cells (PECs) removed. Spleens were homogenized to single-cell suspensions, erythrocytes hypotonically lysed, and cell viability and concentration determined. Cells were serially diluted and plated immediately on indicator MEFs for the purposes of assessing viral reactivation or were cryopreserved in 10% DMSO.

To determine the frequency of cells reactivating lytic viral replication, freshly explanted cells were serially diluted and plated in 96-well tissue culture plates seeded with 10^4^ C57BL6/J MEFs per well. Twenty-four replicates of each cell dilution were plated. Cells were co-cultured for 21 days, and viral reactivation was scored by visual inspection for cytopathic effect (CPE). To control for possible persistent lytic viral replication *in vivo*, the extent of preformed lytic virus in explanted cell populations was quantitated by mechanical disruption of parallel cell samples using 0.5 mm silica beads prior to plating on indicator MEFs. Such mechanical disruption kills >99% of cells but has minimal effect on infectious virus. Under the infection conditions used in these experiments, no significant virus persistence was observed in any genotype of mice infected with MHV68 or ISREΔ viruses.

To determine the frequency of explanted cells that harbored viral genome, cryopreserved cells were thawed, counted, and serially diluted in 96-well thermal cycling plates. Cells were lysed by overnight incubation with proteinase K. Single-copy-sensitivity nested PCR was performed using primers specific for MHV68 ORF72. Amplicons were visualized by agarose gel electrophoresis. Twelve replicates of each cell dilution were analyzed in separate PCR reactions.

### Statistical analysis

Statistical analyses and nonlinear regression were performed using GraphPad Prism 5.0 (GraphPad Software, San Diego, CA). Data from limiting dilution viral genome and viral reactivation assays were fitted to a sigmoidal dose-response curve by nonlinear regression to determine the concentration of explanted cells required to achieve 63% viral DNA-positive PCR reactions or CPE-positive reactivation wells. This cell number was defined according to the Poisson distribution as the reciprocal frequency of viral latency or viral reactivation, respectively, as listed in Table 1.

## Supporting Information

Figure S1 **M2 ISRE specifically binds to nuclear proteins from latently infected mice.** Nuclear proteins harvested from splenocytes of latent C57BL6/J mice were incubated with radiolabeled double-stranded M2 ISRE probe to detect M2 ISRE binding proteins via EMSA (shifted complexes). Unlabeled M2 ISRE or M2 ISREΔ probes were used as competitors at the indicated molar excess relative to labeled probes.
(TIFF)

Figure S2 **Two independently generated ISREΔ mutant viruses, but not MR virus, display enhanced replication** ***in vivo*** **.** 129S2 mice were infected with the indicated viruses and at 9 dpi lungs **(A)** and spleens **(B)** were harvested and infectious virus quantified by plaque assay. Shown are individual organ titers and means (bar) from two to three independent experiments with three mice per group. *p≤0.05, **p≤0.01, ***p≤0.001, by paired t-test comparing ISREΔ or MR to MHV68 in the same host strain at the same time point.
(TIFF)

Figure S3 **Comparison of M2, IFNβ, and IRF2 expression kinetics in the spleen.** Total RNA was harvested from splenocytes of mice infected with MHV68 or ISREΔ at the indicated times post infection. Quantitative RT-PCR was used to detect spliced transcripts of M2 **(A–C),** IFNβ **(D–F)** or IRF2 **(G–I)**. Indicated are the mouse genotypes from which RNA was harvested: 129S2 or IRF2-/- (C57BL6/J background). Expression of all transcripts is shown normalized to internal cellular HPRT mRNA. When comparing these data to Figure 6, note that due to high levels of transcriptionally silent viral DNA (within virions) present during acute infection, M2 expression is not normalized to viral genome levels in this figure. Shown are mean (+/- SEM) from two to three pooled independent experiments with two to three mice per group. *p≤0.05, **p≤0.01, ***p≤0.001, by paired t-test comparing ISREΔ to MHV68 at the same time point (**B**) or IRF2-/- to IRF2+/+ littermates at the same time point (**C**). Statistical comparisons of IFNβ and IRF2 expression (**D–I**) are contained within Figure 2. **N.D**., not detected.
(TIFF)

Figure S4 **Quantitation of latent virus genome frequencies using limiting dilution PCR.** Splenocytes (A–D) and peritoneal cells (E–H) were harvested from 129S2, IFNAR1-/- (129S2 background), IRF2+/+, or IRF2+/- mice latently infected (28–35 dpi) with MHV68, ISREΔ1 or ISREΔ2. IRF2+/+ and IRF2-/- mice were littermates. The frequency of latently infected cells was quantified using limiting dilution, nested PCR for viral genome as discussed in Methods. Viral genome frequency is interpolated from the percentage of 12 replicate PCR reactions, initiated with the indicated number of splenocytes, that are positive for the viral DNA amplicon. Shown is the mean (+/- SEM) of at least three independent experiments with three to five mice per group. Dashed line indicates the point of 63% Poisson distribution generated by non-linear regression used to calculate the frequency of cells harboring viral genome (Table 1). *p≤0.05 by Wilcoxon matched pairs test.
(TIFF)

Protocol S1 **Chromatin Immunoprecipitation (ChIP).**
(DOC)

Protocol S2 **qRT-PCR for cellular and viral transcript quantitation.**
(DOC)
