# Supplementary figures and images for: A Gammaherpesvirus Cooperates with Interferon-alpha/beta-Induced IRF2 to Halt Viral Replication, Control Reactivation, and Minimize Host Lethality
Source: PLoS Pathog. 2011 Nov 17;7(11):e1002371. doi: 10.1371/journal.ppat.1002371 (PMC3219715; doi:10.1371/journal.ppat.1002371)

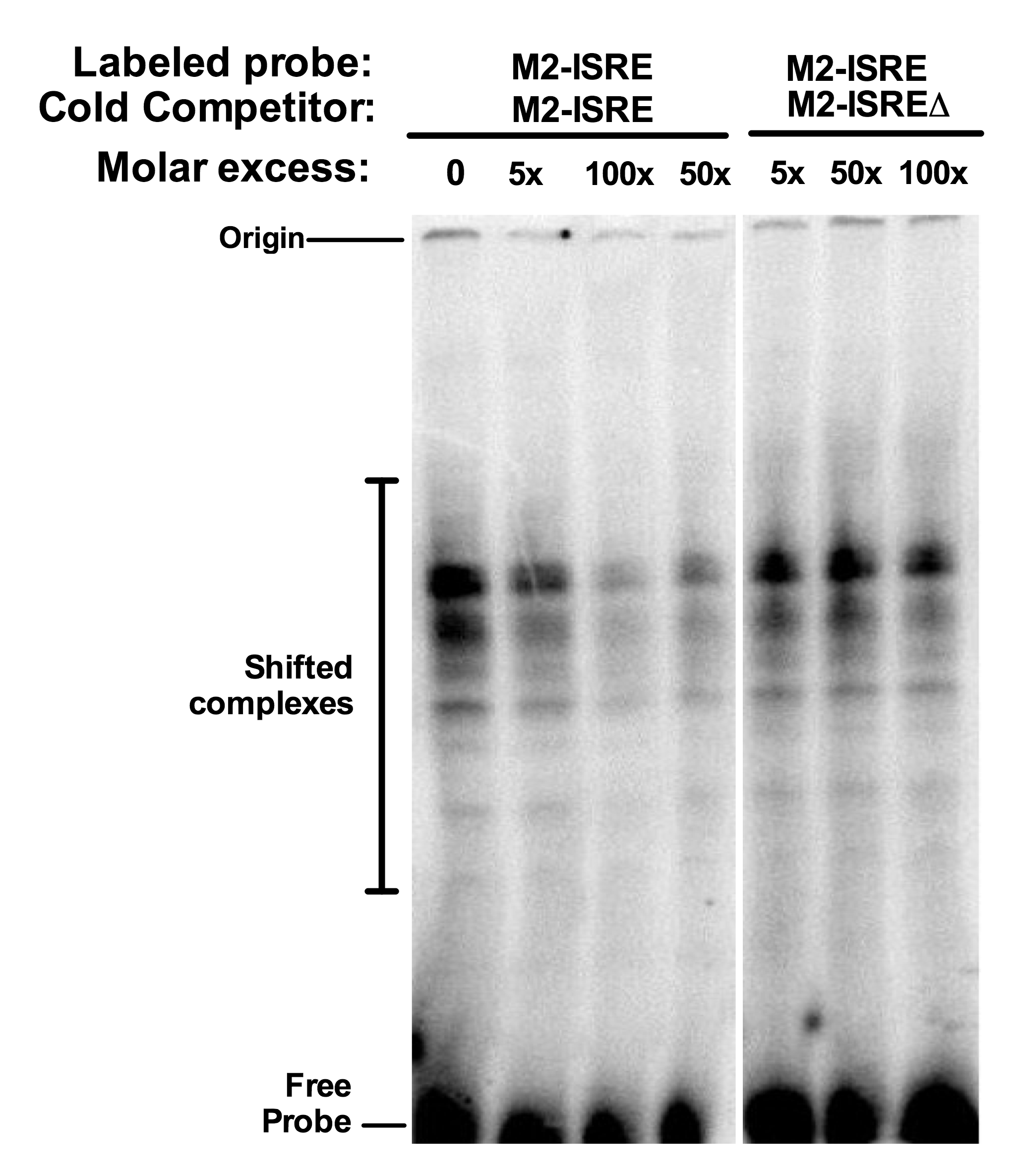

Supplement: Figure S1 — M2 ISRE specifically binds to nuclear proteins from latently infected mice. Nuclear proteins harvested from splenocytes of latent C57BL6/J mice were incubated with radiolabeled double-stranded M2 ISRE probe to detect M2 ISRE binding proteins via EMSA (shifted complexes). Unlabeled M2 ISRE or M2 ISREΔ probes were used as competitors at the indicated molar excess relative to labeled probes. (TIFF) [file ppat.1002371.s001.tif]

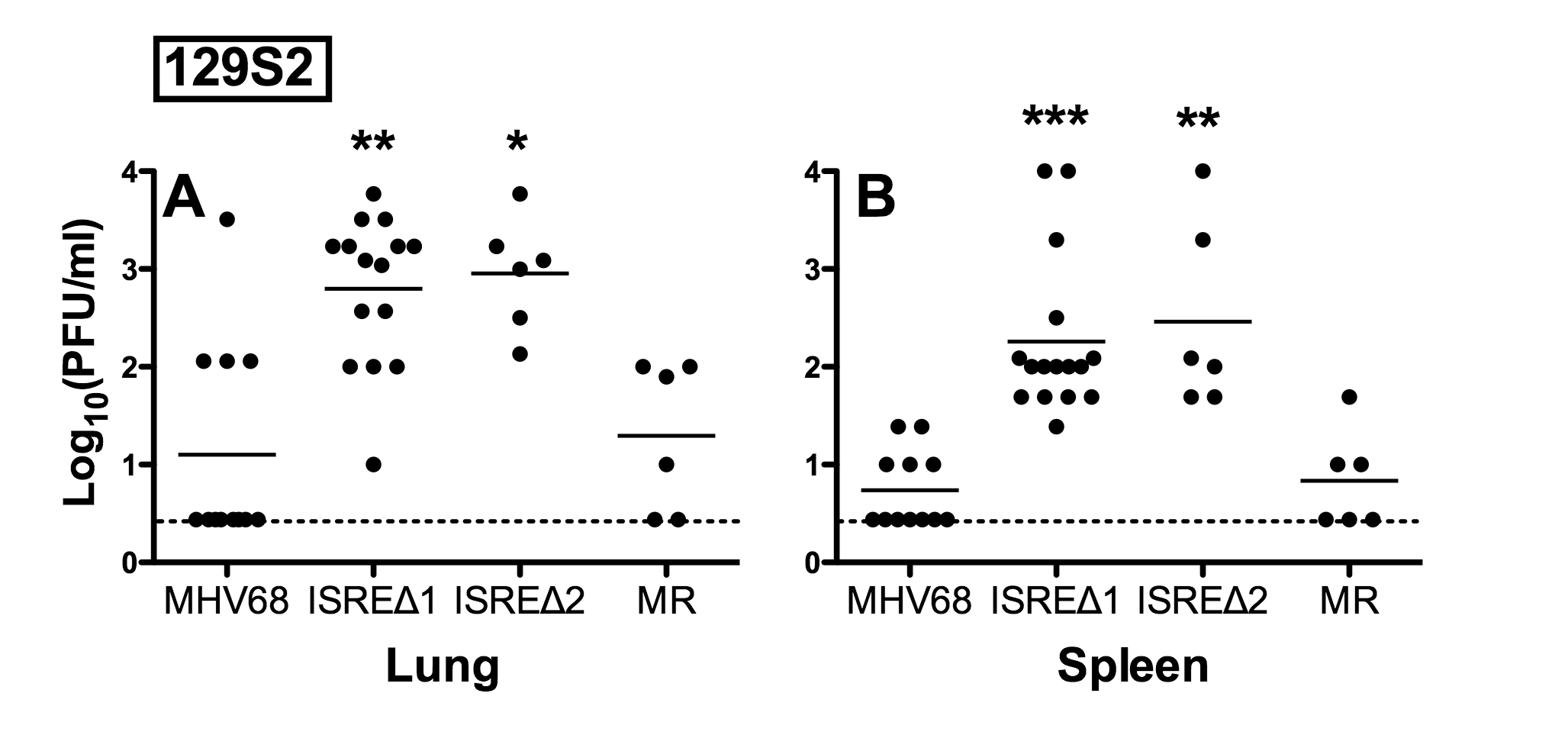

Supplement: Figure S2 — Two independently generated ISREΔ mutant viruses, but not MR virus, display enhanced replication in vivo . 129S2 mice were infected with the indicated viruses and at 9 dpi lungs (A) and spleens (B) were harvested and infectious virus quantified by plaque assay. Shown are individual organ titers and means (bar) from two to three independent experiments with three mice per group. *p≤0.05, **p≤0.01, ***p≤0.001, by paired t-test comparing ISREΔ or MR to MHV68 in the same host strain at the same time point. (TIFF) [file ppat.1002371.s002.tif]

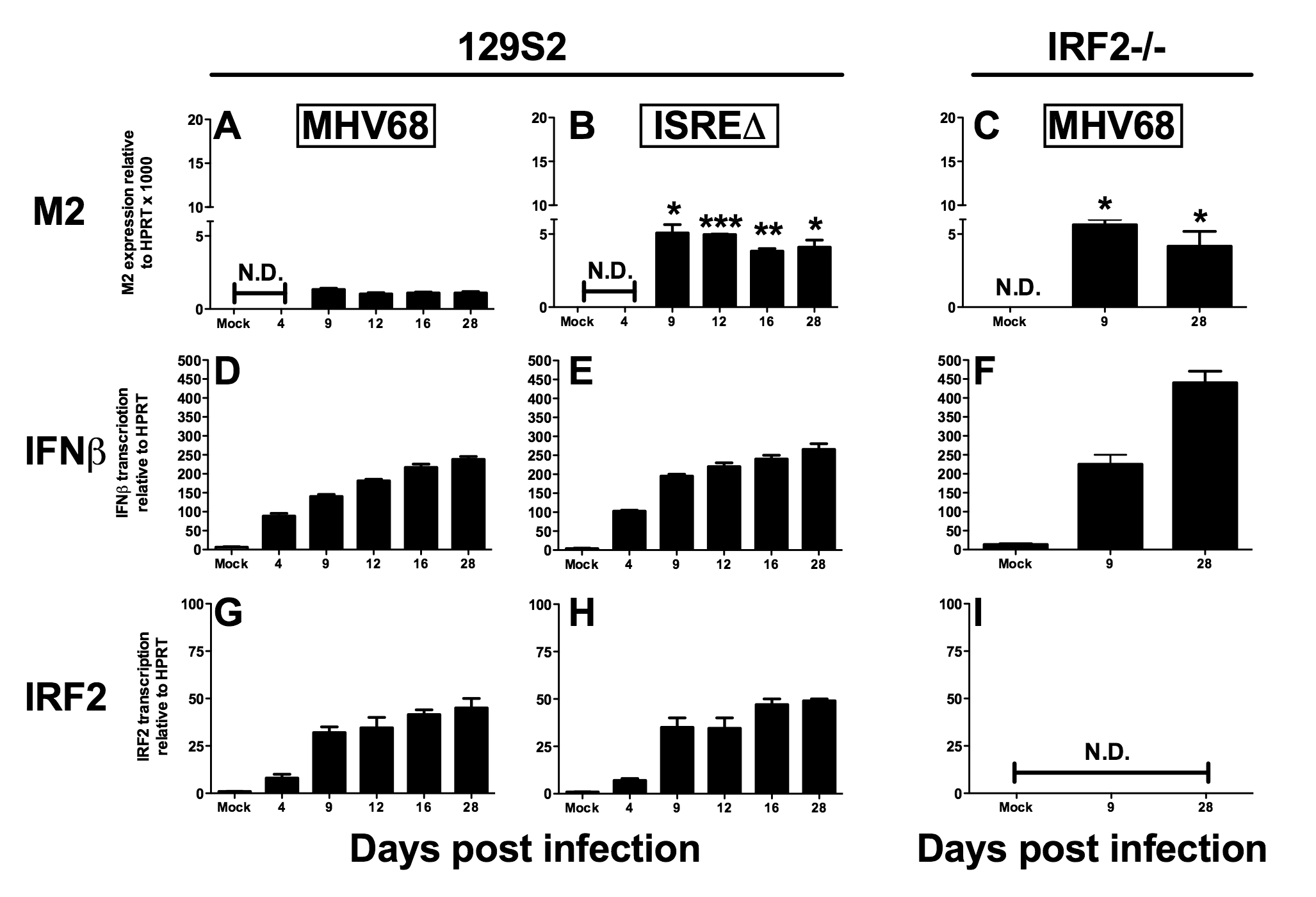

Supplement: Figure S3 — Comparison of M2, IFNβ, and IRF2 expression kinetics in the spleen. Total RNA was harvested from splenocytes of mice infected with MHV68 or ISREΔ at the indicated times post infection. Quantitative RT-PCR was used to detect spliced transcripts of M2 (A–C), IFNβ (D–F) or IRF2 (G–I). Indicated are the mouse genotypes from which RNA was harvested: 129S2 or IRF2-/- (C57BL6/J background). Expression of all transcripts is shown normalized to internal cellular HPRT mRNA. When comparing these data to Figure 6, note that due to high levels of transcriptionally silent viral DNA (within virions) present during acute infection, M2 expression is not normalized to viral genome levels in this figure. Shown are mean (+/- SEM) from two to three pooled independent experiments with two to three mice per group. *p≤0.05, **p≤0.01, ***p≤0.001, by paired t-test comparing ISREΔ to MHV68 at the same time point (B) or IRF2-/- to IRF2+/+ littermates at the same time point (C). Statistical comparisons of IFNβ and IRF2 expression (D–I) are contained within Figure 2. N.D., not detected. (TIFF) [file ppat.1002371.s003.tif]

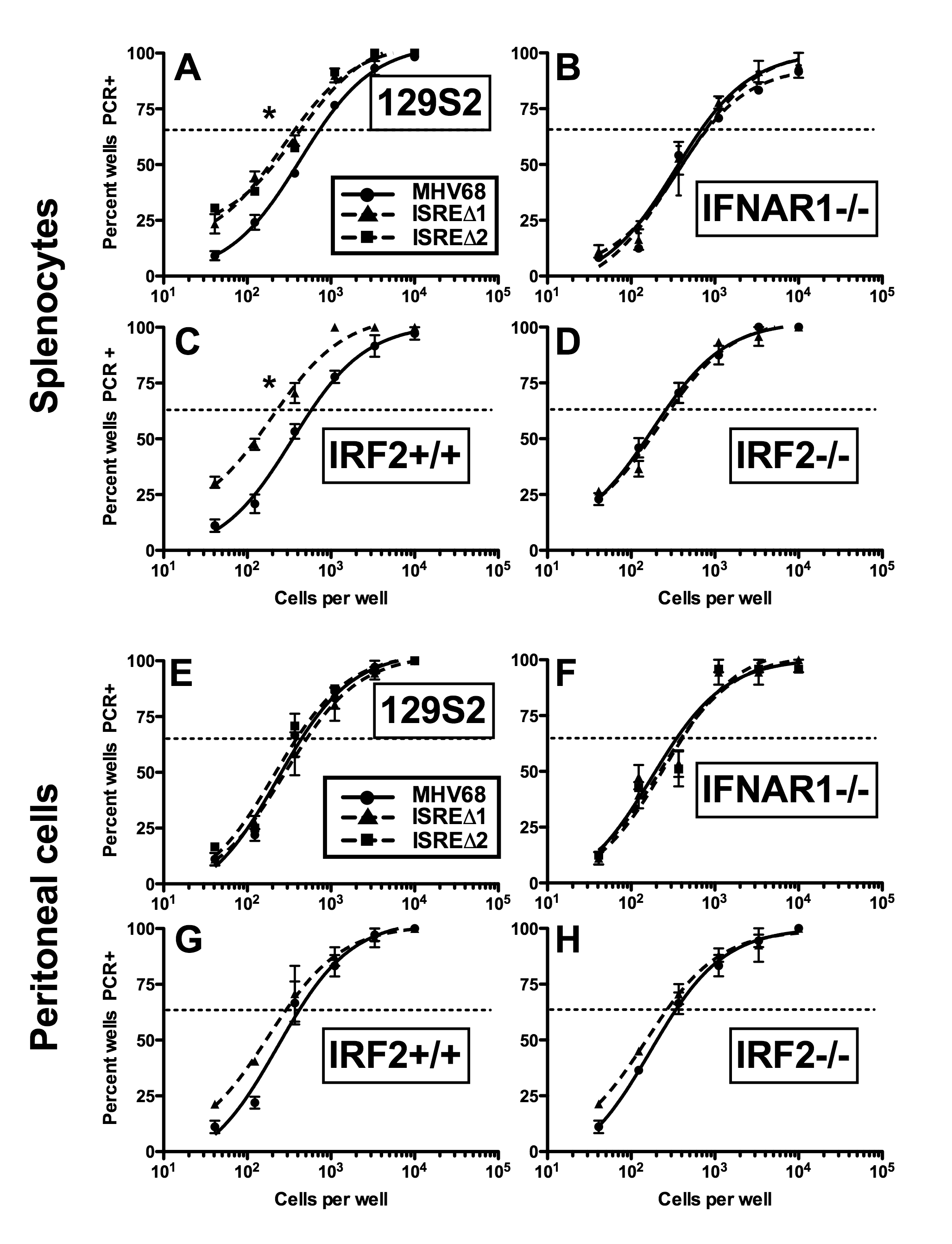

Supplement: Figure S4 — Quantitation of latent virus genome frequencies using limiting dilution PCR. Splenocytes (A–D) and peritoneal cells (E–H) were harvested from 129S2, IFNAR1-/- (129S2 background), IRF2+/+, or IRF2+/- mice latently infected (28–35 dpi) with MHV68, ISREΔ1 or ISREΔ2. IRF2+/+ and IRF2-/- mice were littermates. The frequency of latently infected cells was quantified using limiting dilution, nested PCR for viral genome as discussed in Methods. Viral genome frequency is interpolated from the percentage of 12 replicate PCR reactions, initiated with the indicated number of splenocytes, that are positive for the viral DNA amplicon. Shown is the mean (+/- SEM) of at least three independent experiments with three to five mice per group. Dashed line indicates the point of 63% Poisson distribution generated by non-linear regression used to calculate the frequency of cells harboring viral genome (Table 1). *p≤0.05 by Wilcoxon matched pairs test. (TIFF) [file ppat.1002371.s004.tif]
