# Supplementary material for: A Gammaherpesvirus Cooperates with Interferon-alpha/beta-Induced IRF2 to Halt Viral Replication, Control Reactivation, and Minimize Host Lethality
Source: PLoS Pathog. 2011 Nov 17;7(11):e1002371. doi: 10.1371/journal.ppat.1002371 (PMC3219715; doi:10.1371/journal.ppat.1002371)
Supplement: Protocol S1 — Chromatin Immunoprecipitation (ChIP). (DOC) [file ppat.1002371.s005.doc]

**Protocol S1**

**Chromatin Immunoprecipitation (ChIP)**

Splenocytes (6x10^7^) from latently-infected 129S2 mice were fixed in 1% formaldehyde in protein-free DMEM for 8 minutes, washed in PBS, and sheared in a total volume of 0.5 ml buffer (0.2 M NaCl, 1 mM EDTA, 0.5 mM EGTA, 10 mM Hepes, pH 7.5) using a Misonix S3000 Sonicator (10 second bursts for five, one min cycles). Resulting chromatin had an average length of 500-1000 base pairs. Chromatin from 9X10^6^ cells was diluted in lysis buffer (50 mM Hepes pH 7.5, 140 mM NaCl, 1 mM EDTA, 1% Triton X-100, 0.1% sodium deoxycholate) with protease inhibitor cocktail (Sigma-Aldrich) to a final concentration of 2.4X10^4^ cell equivalents/μl. Diluted samples were mixed with 0.05 ml lysis buffer-equilibrated protein A/G Plus-sepharose beads (Santa Cruz Biotech) and incubated on an end over end rotator for 1 hour at 4^0^C. Following brief centrifugation (300 x *g*, 5 min, RT), 10% (~9X10^5^ cell equivalents) of the supernatant was removed as input DNA PCR control template, and the remaining pre-cleared chromatin was incubated with two μg of IRF2 antisera (H229X, Santa Cruz Biotech) or no antibody as control. Protein A/G Plus-sepaharose beads (0.05 ml) were added after one hour and incubated overnight at 4^0^C. Antibody-bound chromatin was precipitated at RT and mixed with 0.7 ml lysis buffer for 3 min in an end over end rotator. Pellets were subject to sequentially washing in 0.7 ml lysis buffer + 0.5 M NaCl and 0.7 ml ChIP wash buffer (10 mM Tris pH 8, 250 mM LiCl, 0.5% NP-40, 1 mM EDTA), and 0.7ml TE, then eluted from the beads in 0.2 ml ChIP elution buffer (1% SDS, 10 mM EDTA, 50 mM Tris-Cl, Ph 7.5) at 65° C for 10 minutes. Resulting supernatants were protease K treated (two hours, 42° C) and crosslinks reversed by heating for 6 hours at 65° C. DNA was phenol/chloroform extracted, ethanol precipitated, and amplified using PCR primers as described below. PCR parameters used were 95^0^C for 2 min, 40 cycles of 95^0^C for 30 s, 66^0^C for 30 s, 72^0^C for 30 s, followed by 72^0^C for 2 min and hold at 4^0^C. Positive control input PCR reactions contained chromatin from approximately 9x10^5^ cells; immunoprecipitations were initiated with 8x10^6^ cell equivalents of sheared chromatin and 1/10^th^ of resulting reverse-crosslinked immunoprecipitated DNA was used for each PCR reaction. Amplicons were resolved by agarose gel electrophoresis. Negative control (no antibody, or irrelevant rabbit antiserum) immunoprecipitated chromatin yielded no amplicons for any primer set (not shown).

| **Primers used for ChIP** | | | |
| --- | --- | --- | --- |
| **Amplicons numbered according to Figure 1D, left to right)** |  | **Primer sequence** | **Viral genomic coordinates amplified** |
| Experiment 1 | | | |
| Amplicon 1 | Sense | 5’-TAG AGT GGC AGG CCA ACA TAG CCA-3’ | 913-1216 |
|  | Antisense | 5’- TGA GCC TGC CGC TCT ACC AAT-3’ |
| Amplicon 2 | Sense | 5’-TTG TTT CTG GTG CAG AAC TGT CTC CTG-3’ | 2152-2512 |
|  | Antisense | 5’-ACC AGA TCC TGG AGA ACC TGG TTC TAA-3’ |
| Amplicon 3 | Sense | 5’-ACC GCT ACA ATA CTG CGT GGT CTT TA -3’ | 3009-3289 |
|  | Antisense | 5’-AGG CTT AGG ACT GCT GCC CAG GA-3’ |
| Amplicon 4 | Sense | 5’-TGG GAA GAG TCT GTT GAG TGG CCG CGT-3’ | 3749-3988 |
|  | Antisense | 5’-ACA CGC CTG GAA GGA GAA GCG T-3’ |
| Amplicon 5 | Sense | 5’-AGG GCT CGC TGG GAC GGT AGT-3’ | 4492-4737 |
|  | Antisense | 5’- TGT GTG TGG TCG AGA CTG GAG GTT-3’ |
| Amplicon 6 | Sense | 5’- TGC GTG GCC GGG AAG TCT GT-3’ | 5226-5518 |
|  | Antisense | 5’- AGG AGA GAC CCG GAA GGT GGG T-3’ |
| Amplicon 7 | Sense | 5’-TGG GTC AAA ACC ACC TGA CTC GTA TCT-3’ | 5976-6352 |
|  | Antisense | 5’- TGG CCT GGA TTC TCT CAC TTC ACC T-3’ |
| Amplicon 8 | Sense | 5’-ACA TTG TCA TCA GAC AGA CCA AAG GTG TA-3’ | 6776-7113 |
|  | Antisense | 5’-ATC CAA GCC CAC TGT CTG ACA CCA-3’ |
| Amplicon 9 | Sense | 5’-TCC TAC GAT TGC AGA CCA CCA ATA A-3’ | 8132-8480 |
|  | Antisense | 5’-AGA TCA TCC CTG ATT GCC CAA TAG AA-3’ |
| Experiments 2 and 3 | | | |
| Amplicon 2 | Sense | 5’-CCT GCA GGA CAA CTT TAA TGC CAC A -3’ | 2175-2481 |
|  | Antisense | 5’-AGC ATT AAA GTC TGT GAG TCG AAC CAC A-3’ |
| Amplicon 5 | Sense | 5’-ACT TGG CCT AAG ATC CAG GGC TC-3’ | 4476-4727 |
|  | Antisense | 5’- CGA GAC TGG AGG TTC CAA CTG CCT-3’ |
