# Supplementary material for: A Gammaherpesvirus Cooperates with Interferon-alpha/beta-Induced IRF2 to Halt Viral Replication, Control Reactivation, and Minimize Host Lethality
Source: PLoS Pathog. 2011 Nov 17;7(11):e1002371. doi: 10.1371/journal.ppat.1002371 (PMC3219715; doi:10.1371/journal.ppat.1002371)
Supplement: Protocol S2 — qRT-PCR for cellular and viral transcript quantitation. (DOC) [file ppat.1002371.s006.doc]

**Protocol S2**

**qRT-PCR for cellular and viral transcript quantitation.**

RNA was harvested from splenocytes using Trizol reagent (Invitrogen) followed by RNA cleanup (Qiagen) and DNAse treatment (Ambion). RNA (1.5 μg) was reverse transcribed to cDNA using random primers and Superscript II (Invitrogen). Ten percent of the reverse transcription reaction was subjected to quantitative PCR using Fast Start Sybr green ROX (Roche) and oligonucleotide primers (below) using an ABI 7300. For detection of M2, HPRT, IRF2, and IFNβ transcripts, oligonucleotide primers were designed to span exon-intron junctions . For detection of HPRT control and viral genes M2, M3, M9 the cycling parameters were as follows:

95^0^C for 15 min;

40 cycles of:

95^0^C for 30 s,

59^0^C for 60 s,

72^0^C for 30 s;

1 cycle of:

95^0^C for 15 s,

55^0^C for 60 s,

95^0^C for 15 s.

For detection of host IFNβ and IRF2 cycling parameters used were modified as described to the following:

95^0^C for 15 min;

40 cycles of:

95^0^C for 30 s,

56^0^C for 30 s,

72^0^C for 60 s;

1 cycle of:

95^0^C for 15 s,

55^0^C for 60 s,

95^0^C for 15 s.

Transcript expression was analyzed using the comparative C_T_ method as recommended by Applied Biosystems. For host gene expression in Figure 2, the following equations were used to determine ΔC_T_, ΔΔC_T_, and fold induction over controls:

Normalization to housekeeping gene (HPRT), ΔC_T_:

ΔC_T_= C_T(experimental transcript)_-C_T(HPRT)_.

Change in normalized transcripts in experimental relative to control, ΔΔC_T_:

ΔΔC_T_= ΔC_T(experimental transcript)_- ΔC_T(control)_.

Fold induction of host genes in infected mice was calculated as:

2^-(ΔΔC^_T_^)experimental transcript^

Uninfected wildtype mice served as control for this analysis.

Two methods were used for analysis of viral transcript levels:

- For kinetic analysis over time (Figure S3), gene expression levels were expressed as absolute level relative to HPRT using the following equations:

Normalization to housekeeping gene (HPRT), ΔC_T_:

ΔC_T_= C_T(experimental transcript)_-C_T(HPRT)_.

Absolute gene expression relative to HPRT = 2^(-ΔCT)^.

Due to the high levels of transcriptionally-silent viral genomes present during acute infection, data in Figure S3 are not normalized to viral genome copy number.

- During latent infection (Figure 6): First, analysis was performed as in Figure S3. To control for different numbers of latently infected cells present in some samples (Table 1), data were then normalized to viral DNA copy number present as described below, using the following equation:

Genome normalized viral transcript =

(2^-(ΔC^_T_^)viral transcript^) x (2^-(ΔΔC^_T_^)viral DNA copy, control^/2^-(ΔΔC^_T_^)viral DNA copy, experimental)^).

| **Transcripts** |  | **Primer sequence** |
| --- | --- | --- |
| M2 | Sense | 5’-CTGATCACATGGCACTTGGCCTAA-3’ |
|  | Antisense | 5’-CTCAAGCTGCTTCCTTAGCCAGTCTC-3’ |
| M3 | Sense | 5’-AGCCCACTGTCTGACACCAGC-3’ |
|  | Antisense | 5’-ATTCACTGGTGTGCCACCCAC-3’ |
| M9 | Sense | 5’-TCCAGACTTGTCAGGGCCCA-3’ |
|  | Antisense | 5’-TATGGAAAGGACAGTATTGGCAAAGAC-3’ |
| HPRT | Sense | 5’-AAGCAGTACAGCCCCAAAAT-3’ |
|  | Antisense | 5’-TTTGGCTTTTCCAGTTTCACT-3’ |
| IFN-β | Sense | 5’- AGATCAACCTCACCTACAGG-3’ |
|  | Antisense | 5’- TGGAGTTCATCCAGGAGA-3’ |
| IRF2 | Sense | 5’- GGACTCAGCAGCTCTACCCTA-3’ |
|  | Antisense | 5’- CAGTTGAGTCATCTTTGGGGC-3’ |
| **Viral or cellular DNA copies** |  |  |
| GAPDH | Sense | 5’- CCTGCACCACCAACTGCTTAG -3’ |
|  | Antisense | 5’- GTGGATGCA GGGATGATGTTC -3’ |
| ORF-72 | Sense | 5’-TGTCAGCTGTTGTTGCTCCT-3’ |
|  | Antisense | 5’-CTCCGTCAGGATAACAACGTCT-3’ |

and LPS-hyporesponsive macrophages. Infection and immunity 63: 601-608.

**Quantitative PCR for viral genome.**

Detection of viral genome copy number was performed by quantitative PCR for the viral ORF72 genomic locus . Harvested splenocytes were treated with erythrocyte lysis buffer and frozen in DMEM/10 containing 10% DMSO. Quick thawed samples were washed twice with PBS followed by addition of proteinase K in lysis buffer (10 mM Tris pH 8.5, 5 mM EDTA, 200 mM NaCl, 0.2% SDS). Samples were digested overnight (~12 hours) at 56^0^C. Relative copy numbers of ORF72 and GAPDH DNA were determined using 1.5 μg of each sample under the following conditions:

95^0^C for 15 mins;

50 cycles of:

95^0^C for 15 s,

60^0^C for 30 s,

72^0^C for 30 s;

1 cycle of:

95^0^C for 15 s,

55^0^C for 60 s,

95^0^C for 15 s.

**References**

1. Husain SM, Usherwood EJ, Dyson H, Coleclough C, Coppola MA, et al. (1999) Murine gammaherpesvirus M2 gene is latency-associated and its protein a target for CD8(+) T lymphocytes. Proc Natl Acad Sci U S A 96: 7508-7513.

2. DeZalia M, Speck SH (2008) Identification of closely spaced but distinct transcription initiation sites for the murine gammaherpesvirus 68 latency-associated M2 gene. J Virol 82: 7411-7421.

3. Barber SA, Fultz MJ, Salkowski CA, Vogel SN (1995) Differential expression of interferon regulatory factor 1 (IRF-1), IRF-2, and interferon consensus sequence binding protein genes in lipopolysaccharide (LPS)-responsive

4. Gray KS, Allen RD, 3rd, Farrell ML, Forrest JC, Speck SH (2009) Alternatively initiated gene 50/RTA transcripts expressed during murine and human gammaherpesvirus reactivation from latency. Journal of virology 83: 314-328.
